# Energy-Efficient Cluster-Based Data Collection by a UAV with a Limited-Capacity Battery in Robotic Wireless Sensor Networks

**DOI:** 10.3390/s20205865

**Published:** 2020-10-16

**Authors:** Omer Melih Gul, Aydan Muserref Erkmen

**Affiliations:** Department of Electrical and Electronics Engineering, Middle East Technical University (METU), Cankaya, 06800 Ankara, Turkey; aydan@metu.edu.tr

**Keywords:** cluster-based routing, robotic network, energy efficient routing, unmanned aerial vehicle (UAV), wireless sensor network (WSN)

## Abstract

In this work, our motivation focuses on an energy-efficient data collection problem by a mobile sink, an unmanned aerial vehicle (UAV) with limited battery capacity, in a robot network divided into several robot clusters. In each cluster, a cluster head (CH) robot allocates tasks to the remaining robots and collects data from them. Our contribution is to minimize the UAV total energy consumption coupled to minimum cost data collection from CH robots by visiting optimally a portion of the CH robots. The UAV decides the subset of CH robots to visit by considering not only the locations of all CH robots but also its battery capacity. If the UAV cannot visit all CH robots, then the CH robots not visited by the UAV transmit their data to another CH robot to forward it. The decision of transmission paths of transmitting robots is included in the cost optimization. Our contribution passes beyond the existing paradigms in the literature by considering the constant battery capacity for the UAV. We derive the optimal approach analytically for this problem. For various numbers of clusters, the performance of our strategy is compared with the approach in the close literature in terms of total energy consumed by CH robots, which affects network lifetime. Numerical results demonstrate that our strategy outperforms the approach in the close literature.

## 1. Introduction

### 1.1. Motivation

For the last two decades, robotics and wireless sensor networks (WSN) have been very well-investigated separately and so they are very well-known fields. On the other hand, there exist many new opportunities and research directions at the junction of these research fields which combines robot networks and WSNs in one single hybrid network, a robotic wireless sensor network, where robot networks cooperate with WSN to relieve individual disadvantages. Distributing the sensor nodes randomly in the WSN may cause some of the nodes to be located outside of communication range of their neighbors or located in an area outside its node task. A mobile robot can help reposition the nodes in optimal locations, which improves operation, lifetime, and energy efficiency of the network greatly [1]. Robotics can assist in WSNs by servicing the network, in ways such as repositioning nodes, replacing broken nodes, and recharging batteries. The work [2] investigates the problems of robot task allocation and fulfillment to optimally serve WSNs such as recharging batteries and replacing broken or depleted nodes via single-task and multi-task robots.

In [3], the robot replaces the sensors which almost deplete their energy and send out a help localizing signal to it by navigating the WSN based on received signal strength indication (RSSI) from the neighbor nodes and following the route determined by the WSN. Moreover, the robot can help collect data from sensors and fuse these data [4]. The robots can also be used as data mules, which is investigated as the traveling salesman problem in [5] and with the multiple robot case in [6]. However, data muling can cause very long waiting times in transporting data; therefore, the work [7] proposes a clustering method among the data mules to save time. The robot can be used to localize sensors in WSN [8].

Secondly, main applications of WSNs in robotics can be considered as path sensing, mapping, planning, and localization of robots. The WSN help the robots choose the optimal path and avoid dangerous areas [9]. In [10], a modified SLAM algorithm is used for robots. The work [11,12] use particle filters instead of Kalman filters in SLAM algorithm. Beside these, WSNs can provide many tools for coordinating multiple robots and swarm robotics [13]. Using robotics with WSNs makes it easy to share real-time sensor data. Combining these technologies makes sense for a large number of applications. Some applications of WSN using robotics can be listed as military use, weather forecasting, health care, and transport monitoring. Besides these, energy harvesting and low-power WSN may be another possible application area. Furthermore, using both robotics and WSNs with internet in cyber-physical systems (CPS) leads to the Internet of Robotic Things (IoRT), which has brought several changes in various domains that cover several applications in challenging environments. For example, IoRT systems can be used in manufacturing industries to execute difficult tasks such as assembling, packaging, welding, managing quality control, and so on autonomously and remotely [14].

For the last two decades, numerous papers have investigated the energy-efficient data collection problem with a static sink, a fusion center, which collects data from sensor nodes. In these papers, the network is divided into clusters where the cluster head node collects data from the other nodes and send the data to the FC directly or indirectly (forward the data to another cluster head node). The aim is to reduce the energy consumption of the network and thus increase the network lifetime. Although several papers have proposed efficient algorithms to reduce the energy consumption for the problem with static sink, for less energy consumption of cluster head nodes, UAV can be used to collect data from the cluster head nodes and to bring the data to the fusion center. Using UAV has the following advantages. It can access the locations which people or land vehicles can only access with difficulty and risk. UAV can be used when it is expensive and impractical to rent manned aerial vehicles [15]. Therefore, researchers have recently investigated the problem with a mobile sink instead of the static sink in order to reduce the total energy consumption of the network more. In the related literature considering the problem with the mobile sink, there exist many papers which consider the consumed-energy minimization problem as a traveling salesman problem for the cases in which the UAV has sufficient energy to visit all or a constant portion (like half) of CH nodes for data collection. However, to visit a constant portion of the CH nodes, the mobile sink needs a varying battery capacity depending on the locations of the CH robots, which is not practical.

We investigate a data collection problem by a mobile sink, an unmanned aerial vehicle (UAV) with limited battery capacity, in a robot network divided into several robot clusters, each of which has a cluster head (CH) robot, cluster member robots which collect data from the surrounding sensor nodes. Due to the limit on its battery capacity, UAV faces a lack of energy to be able to visit all CH robots depending on their locations and its battery capacity. This battery constraint discriminates our problem from the classical traveling salesman problem where the UAV always has sufficient battery capacity to visit all CH robots. However, the battery capacity of the UAV is constant. Thus, in our problem, the UAV may generally visit a variable number of CH nodes rather than a constant portion of the CH nodes as considered in the recent literature. Our innovative contribution beyond current approaches considers not only variable parametric number of nodes but an optimized CH transmission costs of nonvisited CH robots. If the UAV cannot visit all of CH robots due to the limited battery capacity, then each CH robot not visited by the UAV forwards their data to the CH robots. The UAV aims to minimize total energy consumption of the CH robots by visiting a varying optimized portion of the CH robots. For this purpose, the UAV decides which CH robots to visit by considering not only the locations of CH robots but also its battery capacity.

### 1.2. Our Contributions

The main contributions of this paper can be summarized as follows:

- To the best of our knowledge, this is the first work in which the UAV considers minimizing total energy consumption of the CH robots by visiting a varying optimized portion or all of the CH robots depending on the constant capacity of its battery, whereas the related literature considers the minimum energy path for the mobile sink by visiting all or half of the CH robots/nodes.
- We propose a two-stage solution for this problem. Our method of optimizing the visited subset of CH, taking into account also the transmission of leftout not-visited CH, is derived from a traveling salesman problem when the UAV visits a constant portion of CHs. Our paper will explicitly provide this derivation and then clearly provide the innovative abilities of our approach by removing a varying portion of the CH robots from the path in order to reduce the energy consumption of the UAV below the battery capacity of the UAV. For this purpose, the UAV considers both the locations of CH robots and its battery capacity along with transmission hops in energy usage of the nonvisited CH robots. As a result, the UAV desists to visit the CH robots which consumes less energy for forwarding their data and which the UAV requires more energy to visit.
- We also find optimal data forwarding strategies for all nonvisited CH robots by considering all possible transmission paths in each case. Here, we consider each combination (subset) of CH robots as a separate case in which each CH robot in this combination (subset) of CH robots looks for an optimal transmission path to forward its data to the UAV via hops (via other CH robots).

### 1.3. Organization

The remainder of this paper is organized as follows. In Section 2, we provide the related work about energy-aware, cluster-based routing protocols with both static sink and mobile sink cases. The problem definition along with the system model are given in Section 3. In Section 4, we consider the case in that the UAV has sufficient energy to visit all CH robots for data collection that will help us explicitly derive our novel approach which will be the focus of Section 5 which includes the novelty of this paper. In Section 6, we provide numerical results for total energy consumption of CH robots in different scenarios. Section 7 concludes the paper.

## 2. Related Work

In this section, we provide the related work about energy-aware, cluster-based routing protocols with both static sink and mobile sink cases.

### 2.1. Energy-Aware, Cluster-Based Routing Protocols with Static Sinks

Power consumed by the sensors has a significant influence on the network lifetime. If a sensor node send data directly to the base station, the energy consumed in this transmission is much greater than the energy consumed in inter-sensor communication since the distance between the base station and a sensor is generally much greater than the distance between two neighboring sensors. To deal with this problem, many cluster-based routing protocols have been proposed for the last two decades. These protocols first divide the WSNs into regions—“clusters”—then, a cluster head is selected in each cluster to collect data from the other sensors in its cluster and send data to the base station. To distribute the extra power consumption of being cluster head, each cluster-head sensor leaves this responsibility to another sensor in the cluster after a while.

In the work [16,17], the authors propose a cluster-based protocol, low-energy adaptive clustering hierarchy (LEACH), which makes randomized rotation of cluster-heads to equally distribute the energy consumption among the sensors in the same cluster. LEACH assumes that all sensors are homogeneous, energy-limited, and immobile (static); the base station is far from the sensors. LEACH uses localized coordination for enabling robustness and scalability in dynamic networks. It also makes fusion of the collected data to decrease the size of the data to be sent to the base station and so reduce the power consumption. In LEACH protocol, each cluster head schedules the sensors via TDMA for the intra-cluster data collection and different CDMA is used for inter-cluster traffic to prevent the data transmissions from the interference. LEACH reduces the energy consumption eight times and increase the network lifetime two times compared to the previous protocols of direct transmission, minimum transmission energy, multi-hop routing, and static clustering.

In the work [18], the authors consider that a cluster-head spends more energy than the others while it collects data from cluster members, fuses data to decrease its size, or transmits the aggregated data to a base station. Regarding each sensor as an ant and each cluster as a nest in this work, the authors propose a novel clustering mechanism based on ANTCLUST [19] where clusters are organized in a distributed and energy-efficient way through local communication among neighboring sensor nodes. It is numerically shown that the ANTCLUST based algorithm can collect data from more than 80% of the sensors longer than LEACH by over 25% and it also extends the network lifetime 150% compared with LEACH. In the work [20], the authors propose a cluster-based routing protocol, stable cluster head election (SCHE). SCHE differs from LEACH such that SCHE do not change the cluster heads in each round whereas the traditional LEACH changes the cluster heads in each round. Thus, SCHE reduces energy consumption very much compared with the LEACH (up to 90%).

In the paper [21], the authors propose a cluster head election protocol, advanced LEACH (ALEACH). LEACH makes decisions independent from the energy remained (present energy condition) in sensors, which is an important drawback of LEACH. ALEACH overcomes this problem by introducing two terms, general probability and current probability, in the threshold equation. In the work [22], the authors propose an energy-efficient routing protocol, time-based LEACH (TB-LEACH) which just changes the cluster head election. Thus, it improves the cluster partition and forms uniform and balanced clusters. In the work [23], the authors propose an energy-aware routing protocol, distributed and energy-efficient self organization (DEESO). Electing the cluster head is adjusted to the remained energy in the battery of sensors. It is a completely distributed approach and adaptive channel assignment (ACA) is applied for addressing the on-off mode changes of sensors in the network topology. Thus, it increases the network lifetime and transmits three times more data compared with LEACH.

In the work [24], the authors propose a clustering protocol, LEACH-IMP. This protocol determines the optimal cluster heads by considering the positions of the sensors. By keeping the cluster head constant, LEACH-IMP is much more energy efficient than LEACH. In the work [25], the authors propose an energy-aware routing protocol. Different from LEACH [16] and LEACH-F [17], this protocol uses dynamic round times; in other words, it changes the round times depending on the remained energy of the sensors. Thus, this protocol consumes considerably less energy and increases the network lifetime compared with LEACH and LEACH-F. In the paper [26], the authors propose a new routing protocol, two-step cluster head selection (TSCHS) routing protocol. TSCHS aims to solve an important problem of LEACH, the variability of the number of cluster heads. This protocol has two stages to select the cluster heads. In the work [27], the authors propose an energy-aware routing protocol, modified LEACH (ModLEACH). Different from the LEACH, ModLEACH introduces an efficient cluster head replacement scheme with dual transmission power levels which are used for decreasing interference and collisions. Thus, ModLEACH decreases packet drop ratio.

Although the protocols with static sink give good results, mobile sink visiting cluster heads provide the opportunity to decrease the total energy consumption of the network more and so increase the network lifetime more. Therefore, the protocols with mobile sink have been investigated and preferred more in recent years. We consider the problem with mobile sink as well. In the next subsection, the protocols with mobile sink are surveyed.

### 2.2. Energy-Aware, Cluster-Based Routing Protocols with Mobile Sinks

Collecting data using mobile sink provides an effective solution to the energy-hole problem in WSN, which may be faced in WSNs with static sink where sensor nodes forward their data towards static sink. The approaches using mobile sink for this problem can be divided into two main categories: direct and rendezvous approaches. With direct approaches, the mobile sink collects data from each node based on one hop distance metrics. With rendezvous approach, the mobile sink travels just a limited number of nodes named as rendezvous points (RP) and establishes the local routing for data collection from all other nodes to primary sensor nodes [28]. In our problem, the UAV collects data from the CH robots; therefore, the papers considering the problem with mobile sink are closer to our work than the ones tackling the problem with static sink. In this subsection, we survey the main existing approaches in the related literature considering the data collection problem with mobile sinks.

In [29], an energy-aware path construction (EAPC) policy is proposed for collecting data based on environmental monitoring. With EAPC, the mobile sink chooses a suitable set of locations for collecting data and plans a path for collecting data; then, it starts data collection from the points burdened with data. In terms of energy consumption and network lifetime, EAPC is more efficient than weighted rendezvous points (WRP) policy, which assigns a weight to each sensor and considers the nodes with the highest weight as data collection points [30].

In the paper [31], the whole sensor field is divided into sectors, each of which calculates its members’ weights and selects a cluster head (CH). Member nodes search for the optimal scenario by calculating energy consumption of different routing paths. Then, CHs form connections for intercluster communication via a greedy policy. The proposed algorithm performs better than two algorithms (considering the similar problems), the energy-efficient cluster-based dynamic routing algorithm (ECDRA) and the cluster-chain mobile agent routing (CCMAR). The work [32] considers a network which consists of static sensor nodes distributed uniformly randomly and a mobile sink with unlimited battery capacity. The authors propose the MIEEPB-DT protocol which combines direct transmission (DT) protocol with the mobile sink improved energy-efficient power-efficient gathering in sensor information system-based routing protocol (MIEEPB) [33] for the efficient usage of the limited energy of nodes. MIEEPB-DT performs better than DT and MIEEPB for network lifetime and energy efficiency.

The paper [34] considers a data collection problem in WSNs via a mobile sink with unlimited battery capacity. An energy-efficient trajectory planning (EETP) technique is proposed using multi-objective particle swarm optimization (MOPSO) for balancing the load of rendezvous nodes and shortening the path of the mobile sink. EETP performs much better than WRP [30], CB in terms of energy consumption, and thus network lifetime. The work [35] considers a network where a mobile sink collects data from sensor nodes by using rendezvous points (RP). It has already been proven that meta-heuristics such as particle swarm optimization (PSO) shows a feasible and promising performance for forming the trajectory. The work [35] proposes a PSO-based RPs selection (PSO-RPS) technique which outperforms against the methods in related literature in terms of trajectory length. In [36], a hyperheuristic framework is proposed and it can construct high-level heuristics automatically for path planning by using the genetic algorithm. This algorithm prolongs the network lifetime.

The paper [37] proposes a cluster-based data collection protocol where the optimal cluster heads are selected to reduce energy consumption. The optimal path a mobile sink plans the optimal path by ant colony optimization (ACO) algorithm. The mobile sink with unlimited battery capacity plans an efficient path for data collection along with the cluster centroid. The proposed method is analyzed in terms of lifetime and energy usage. The paper [38] proposes a five-stage solution for the cluster-based routing problem. First, the network is divided into multiple regions by quad tree combined binary tree policy. Secondly, the authors carry out weight-based cluster head selection (WCHS) method to select a cluster head in each partition. Then, it uses a novel pair-based sink relocation scheme (PSRS) for relocating the sink node. After then, the authors execute a destination-oriented directed acyclic graph (DODAG)-based route adjustment by considering three rules. Finally, type-2 fuzzy-based adaptive MAC scheduling is used. This protocol decreases energy consumption up to 20% and so extends the network lifetime up to 30% compared with GR and QDVGDD methods.

The paper [39] proposes a joint density-aware and energy-limited path construction algorithm for data collection, called DEDC, aiming to select as more as possible appropriate anchors under the path length constraint for prolonging the network lifetime. Initially, the proposed DEDC determines the grid size according to the path length constraint, partitions the monitoring region into several grids, and identifies the grids to be balance or unbalance grids. Based on the partitioned grids, the proposed DEDC constructs a regular path and then further adjusts the path segments for these unbalanced grids. The regular path construction and path adjustment aim to construct a path passing through as more as possible anchors for balancing the forwarding loads and prolonging the network lifetime. Performance evaluations reveal that the proposed DEDC outperforms existing data collection mechanisms in terms of energy consumption, network lifetime, and SD energy consumptions.

The paper [40] proposes two data collection policies for cluster-based WSN: (1) WSN-oriented and (2) UAV-oriented. In the WSN-oriented approach, nodes within each cluster member (CM), send information to their cluster head (CH) and for recollection, the UAV visits all CHs. As the UAV visits many CHs, the flight time is increased. In the UAV-oriented approach, all CHs send data from their CMs to a sink node; hence, the UAV only visits this node, reducing the flying time but with a higher system energy cost. To find the most suitable scheme for different monitoring conditions in terms of the average energy consumption and the buffer capacity of the system, the authors develop a mathematical model that considers both the dynamics of the WSN along with the UAV.

In the related literature, the papers investigate the problem without considering any limit on the battery capacity of the mobile sink. They consider the problem with a mobile sink visiting a constant portion of cluster heads like visiting half of them. However, visiting a constant portion of CH nodes requires varying battery capacity of the mobile sink depending on the topology of the network, which is impossible for the UAV (battery capacity of the mobile sink cannot vary). Our paper fills this gap in the literature by considering the finite constant-capacity battery for the UAV. In our innovative approach, by choosing an energy optimally varying portion of the CH robots to visit, the UAV with finite constant-capacity battery aims to minimize total energy consumption of the nonvisited CH robots that will be transmitting data by multiple hops through other nonvisited CH until a visited CH robot. In our work, deciding the subset of CH robots to visit depends on not only the locations of CH robots but also its battery capacity.

## 3. System Model and Problem Definition

This section focuses on defining our problem and generates our system approach. Table 1 summarizes the notation and symbols commonly used in this paper for ease of reference.

### 3.1. Motivation and Problem Definition

We will present here a motivating scenario and formulate the problem based on this motivation. We interchangeably use robot, node, and sensor in the rest of this paper. We consider a robotic network which consists of *M* clusters of mobile robots, an unmanned aerial vehicle (UAV) with limited battery capacity and the fusion center where the UAV charges its battery. The responsibility of the robotic network is to collect data from the sensors which monitors environmental changes such temperature, humidity, noise, etc.

**Figure 1:**
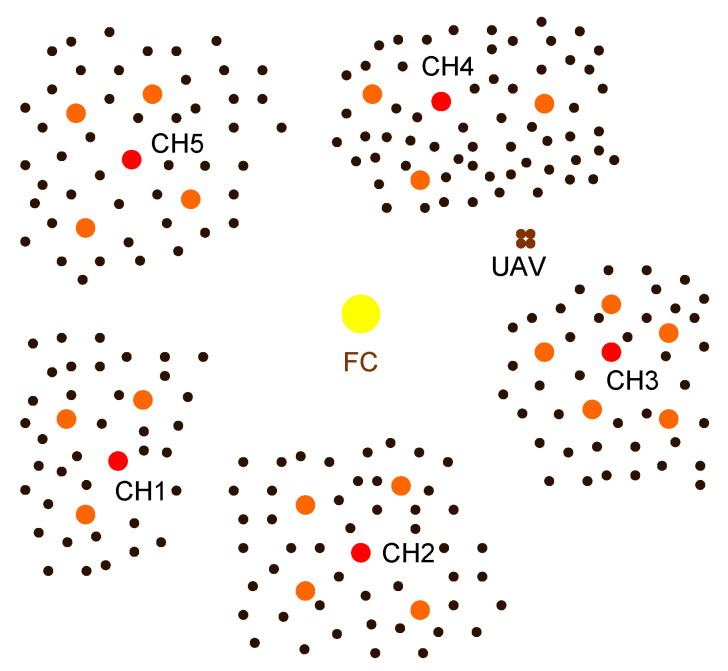
The whole multi-robot system includes a fusion center (FC) and five clusters of robots around FC. Red dots are the cluster head (CH) robots whereas the orange ones are the remaining robots which collects (environmental monitoring) data from the sensors (the black dots) in their cluster and send their data to the CH robot of their cluster. There are 19 robots in total and the UAV in the system. The UAV initially stands on the FC to charge its battery. After the UAV collects data from all CH robots directly or indirectly, it returns to FC to send all data to FC and recharge its battery for the next path.

Each cluster has a cluster head (CH) robot which allocates tasks to the remaining robots in the cluster. The remaining robots execute the tasks assigned to them (such as monitoring the environment and detecting unusual cases) and send the resultant data (obtained using their sensors) to the CH robot in its cluster. The responsibility of a CH robot is to collect data from the robots in its cluster and then transmit data to the UAV directly or indirectly (by sending its data to another CH robot to forward to the UAV). In this network, UAV visits some of the CH robots or all of them depending on their locations and the battery capacity of the UAV. Please see Figure 1. If the UAV cannot visit all of CH robots due to its limited battery capacity, then the CH robots which is not visited by the UAV send their data to one of neighboring CH through multiple hops via other nonvisited CH neighbors.

The index set of all CH robots in the network is denoted by S≜{1,2,⋯,M}. In the multi-robot system, each CH robot collects data from the remaining robots (in its cluster) to aggregate and transmit to the UAV. The battery capacity of the UAV is denoted by *B*.

A CH robot spends significant energy for data aggregation and transmission to the UAV or the neighboring CH robot. If a CH robot moves like the remaining robots in its cluster, then the energy level of the CH robot will decrease below the critical energy level quickly. Falling below the critical level fast causes frequent CH election which is a very time and energy consuming process. It may also cause a CH robot which allocates tasks very efficiently to leave being CH robot a result of inefficient energy consumption due to moving like other robots in the cluster. Hence, it is reasonable to make the following assumption.

**Assumption** **1.** *A CH robot does not move during its CH mission to avoid additional energy consumption.*

**Remark** **1.** *From Assumption 1, we need to focus on the positions of the CH robots and the battery capacity of the UAV. Therefore, the sensors and the robots except CH robots and the UAV will not be shown in the following figures except Figure 1.*

Initially, we aim to focus on the distances for calculating the costs of energy consumption. Therefore, we make the following assumption.

**Assumption** **2.** *Each CH robot sends the same amount of data without latency to the UAV (directly or indirectly by forwarding its data).*

The positions of the CH robots and initial position of the UAV are denoted by using Cartesian coordinates. The position (vertice of the network) of the CH robot *i* (of the cluster *i*) is denoted by $\xi_{i}≜\left(x_{i},y_{i}\right)$. The initial position of the UAV is denoted by $\xi_{0}≜\left(x_{0},y_{0}\right)$. It is assumed that the energy consumption of the UAV is proportional to the distance it travels. To illustrate, the energy consumed by the UAV from CH robot *i* to CH robot *j* is denoted by $E_{UAV}\left(i,j\right)$ and this energy cost is defined as

(1) $$\begin{array}{ccc} E_{UAV}\left(i,j\right) & ≜ & C_{UAV}∥\xi_{i}-\xi_{j}∥\\ = & C_{UAV}\sqrt{{\left(x_{i}-x_{j}\right)}^{2}+{\left(y_{i}-y_{j}\right)}^{2}} \end{array}$$

where $C_{UAV}$ is the constant ratio between the energy consumption of the UAV and the distance it travels, which represents the direct proportionality. On the other hand, it is assumed that the energy consumption (for data transmission) of a CH robot is proportional to the square of the distance between itself and the next hop. (The next hop may be a neighboring CH robot or the UAV depending on the path the UAV for data collection.)

To illustrate, the energy consumed by the CH robot *i* to transmit data to CH robot *j* is denoted by $E_{CH}\left(i,j\right)$ and this energy cost is defined as

(2) $$\begin{array}{ccc} E_{CH}\left(i,j\right) & ≜ & C_{CH}{∥\xi_{i}-\xi_{j}∥}^{2}\\ = & C_{CH}\left[{\left(x_{i}-x_{j}\right)}^{2}+{\left(y_{i}-y_{j}\right)}^{2}\right] \end{array}$$

where $C_{CH}$ is the constant ratio between the energy consumption of a CH robot and the square of distance between it and the next hop (neighboring robot), which represents the quadratic proportionality (From Assumption 2, the amount of data to send is same for each CH robot in a round. Therefore, $C_{CH}$ is constant for each CH robot).

In the related literature, it is assumed that the robots or nodes have sufficient energy to transmit their data to the FC which is the initial position of the UAV. During the path, it is very probable that the UAV may pass through a closer point for each CH robot. In the worst case, the UAV can collect data from some CH robots when it is standing on the FC (the initial position of the UAV). Therefore, we make the following assumption in this work.

**Assumption** **3.** *It is assumed that a CH robot has sufficient energy to transmit its data directly to the UAV when it is at its initial position.*

Data transmission rate while communicating with the UAV is constant for all CH robots because this is more energy efficient from the fact that data transmission rate is logarithmic function of data transmission power.

**Assumption** **4.** *A CH robot transmits data to the UAV or another CH robot with constant rate.*

In other words, if a CH robot *i* consumes $\alpha_{1}$ units energy to transmit $\beta_{1}$ bits and $\alpha_{2}$ units energy to transmit $\beta_{2}$ bits, then a CH robot *i* consumes $\alpha_{1}+\alpha_{2}$ units energy to transmit $\beta_{1}+\beta_{2}$ bits. Depending on the battery capacity of the UAV, it may visit a portion of CH robots instead of all CH robots due to the lack of energy. As this problem considers efficient energy usage of the UAV beside the CH robots, we make the following assumption.

**Assumption** **5.** *The UAV visits each CH robot at most once.*

Under these assumptions, we will define the problem more precisely in the following subsection.

### 3.2. Our Problem Approach Formulation

In this work, the UAV aims to plan such a path that it can complete with the energy in its full battery. Although each CH robot has sufficient energy to transmit their data in one round, it is not desired that the CH robots spend much energy for this data transmission. *The UAV aims to minimize the total energy consumption of the CH robots by planning the path through which it visits the CH robots.*

**Definition** **1** **(Path Set, P).** *Let’s define the path set as the set of all M×(M+1) the linear paths between the positions of the M CH robots and the initial position of the UAV which are denoted by*

(3) $$P≜\{p\left(\xi_{0},\xi_{1}\right),p\left(\xi_{0},\xi_{2}\right),\cdots ,p\left(\xi_{0},\xi_{M}\right),\cdots ,p\left(\xi_{i},\xi_{j}\right),\cdots ,p\left(\xi_{M},\xi_{M-1}\right)\}.$$

*where $p(\xi_{i},\xi_{j})$ is the linear path (edge of the network) from CH robot i to CH robot j if i≠0 and j≠0) and the length of $p(\xi_{i},\xi_{j})$ is equals to $∥\xi_{i}-\xi_{j}∥$. (Notice that $p(\xi_{0},\xi_{j})$ is the linear path (edge of the network) from the initial position of the UAV to CH robot j and $p(\xi_{i},\xi_{0})$ is the linear path from CH robot i to the initial position of the UAV.)*

**Definition** **2** **(Strategy of the UAV, π).** *Let us define the strategy of the UAV as the set of the linear paths followed by the UAV for the data collection, which is a subset of the path set, i.e., π⊆P.*

**Definition** **3** **(Data forwarding strategy of CH robot i, $u_{i}$).** *Let us define the data forwarding strategy of CH robot i as the set of the linear paths followed by the CH robot i for forwarding data to a CH robot on the route (visited by the UAV). Notice that $u_{i}=\emptyset$ for all CH robot i visited by the UAV. The data forwarding strategy is a subset of the path set, i.e., $u_{i}\subseteq P$.*

**Definition** **4** **(The set of data forwarding strategies of CH robots, u).** *Let us define the set of data forwarding strategies of CH robots as the set of all data forwarding strategies of CH robots such that $u=\{u_{1},u_{2},\cdots ,u_{M}\}$, where $u_{i}=\emptyset$ for all CH robot i visited by the UAV.*

**Definition** **5** **(Indicator function).** *The indicator function is a binary function which takes a value of 1 for true event A, i.e.,*

(4) $$I_{\left\{A\right\}}≜\left\{\begin{array}{cc} 1 & \;if\;event\;A\;is\;true\\ 0 & \;if\;event\;A\;is\;false \end{array}\right.$$

**Definition** **6** **(Energy consumption of the UAV under strategy π, $E_{\mathrm{UAV}}^{\pi }$).** *Let us define energy consumption of the UAV under strategy π as the total energy consumed by the UAV whenever the strategy π is applied, i.e.,*

(5) $$E_{UAV}^{\pi }≜\left[\sum_{j=1}^{M}\sum_{i=1}^{M}E_{UAV}\left(i,j\right)I_{\left\{p(\xi_{i},\xi_{j})\in \pi \right\}}\right]$$

From Equation (1), Equation (5) yields

(6) $$E_{UAV}^{\pi }=\left[\sum_{j=1}^{M}\sum_{i=1}^{M}C_{UAV}∥\xi_{i}-\xi_{j}∥I_{\left\{p(\xi_{i},\xi_{j})\in \pi \right\}}\right].$$

**Remark** **2.** *Notice that if a CH robot i is not visited by the UAV, then*

(7) $$\sum_{j=1}^{M}I_{\left\{p\left(\xi_{i},\xi_{j}\right)\in \pi \right\}}=0.$$

**Definition** **7** **(Energy consumption of CH robot *i* under strategy π, $u_{i}$, $E_{i}^{\pi }\left(u_{i}\right)$).** *Using the data forwarding strategy $u_{i}$, a CH robot i not visited by the UAV under strategy π consumes $E_{i}^{\pi }\left(u_{i}\right)$. If a CH robot i is visited by the UAV under strategy π, $E_{i}^{\pi }\left(u_{i}\right)=0$ (no need for data forwarding strategy).*

**Definition** **8** **(Total energy consumption of all CH robots under strategies π, u, $E_{\mathrm{ACH}}^{\pi }\left(u\right)$).** *Let us define energy consumption of all CH robots under strategy π as the total energy consumed by all CH robots whenever the strategy π is applied, i.e.,*

(8) $$E_{ACH}^{\pi }\left(u\right)≜\sum_{i=1}^{M}\left[E_{i}^{\pi }\left(u_{i}\right)\left(1-\sum_{j=1}^{M}I_{\left\{p\left(\xi_{i},\xi_{j}\right)\in \pi \right\}}\right)\right].$$

Under Assumptions 1–5, we formulate our approach precisely as in Problem 1.

**Problem** **1.** *Minimizing total energy consumption of CH robots via an limited-battery UAV*

$$\begin{array}{cccc} \; & \underset{\pi \subseteq P}{min}\; & \; & E_{ACH}^{\pi }\left(u\right)\\ \; & s.t.\; & \; & E_{UAV}^{\pi }\leq B \end{array}$$

## 4. Sufficient Battery Capacity to Visit All CH Robots

In this section, we consider the case in that the UAV has sufficient energy to visit all CH robots for data collection. first, we will consider the problem as a traveling salesman problem and then look for the shortest path. Thus, we can obtain a lower bound for the amount of energy which the UAV needs to be able to visit all CH robots.

**Definition** **9** **(Minimum battery capacity for the UAV to visit all CH robots, $B_{\mathrm{TSP}}$).** *The energy required for visiting all CH robots under the optimum strategy in the traveling salesman problem is the minimum battery capacity for the UAV to visit all CH robots.*

To find this minimum battery capacity to visit all CH robots, we consider the problem as the classical traveling salesman problem. To find a robust optimal solution for the traveling salesman problem at hand, we consider the following remark.

**Remark** **3.** *In a multirobot network system, one actually deals with hundreds of robots at most, which implies that at most tens of CH robots exist in the network. Roughly speaking, square root of the number of robots can be taken in this process to find the number of CH robots approximately.*

From Remark 3, we can find optimal solution for the TSP by using common techniques in the literature, like the genetic algorithm (GA) or the particle swarm optimization algorithm (PSO). In this paper, we use GA to solve TSP. Thus, for our network including the UAV and CH robots, we apply GA as optimal solution in case the UAV has sufficient battery capacity to visit all CH robots for data collection.

## 5. Our Novel Approach: Total Energy Minimization Problem

The main novelty of the paper comes from the case investigated in this section. Although there exist many papers considering the first case described in Section 4, to date, there exists no paper considering the finite-capacity battery considered in this section. (In the related literature, the second case is considered as visiting a constant portion of the cluster heads, like visiting half of them. However, for visiting constant portion of the cluster heads, the UAV needs a varying battery capacity depending on the locations of the CH robots, which is not practical.).

In order to motivate our novelty, the UAV has insufficient energy to visit all CH robots. In this case, by choosing an energy optimally varying portion of the CH robots to visit, the UAV aims to minimize total energy consumption of the CH robots not visited by the UAV that will be transmitting data by multiple hops through other nonvisited CH until a visited CH node. Therefore, we will focus on minimizing energy consumption of each CH robot which is not visited by the UAV under strategy π. In the next subsection, we will investigate optimal strategies for the nonvisited CH robots to forward their data to another CH robot until a visited CH robot.

### 5.1. Optimal Data Forwarding Strategy of CH Robots

If a CH robot *i* is visited by the UAV, then there is no need for the data forwarding strategy and so $u_{i}=\emptyset$. On the other hand, if a CH robot *i* is not visited by the UAV, then it should look for the shortest path to each CH robot visited by the UAV and take the minimum of all shortest paths. From Equation (2), the squares of the distances between the neighboring CH robots are considered to calculate the shortest path between the CH robots. Thus, we can derive optimal data forwarding strategy for each CH robot. Please notice that each of the nonvisited CH robots considers each of the visited CH robots visited by the UAV as a possible target CH robot to find the optimal data forwarding strategy for itself.

**Definition** **10** **(Minimum energy consumption of CH robot *i* not visited under strategy π, $\gamma_{i}^{\pi }$).** *Let us define minimum energy consumption of CH robot i not visited by the UAV under strategy π, i.e.,*

(9) $$\gamma_{i}^{\pi }=\min_{u_{i}}E_{i}^{\pi }\left(u_{i}\right)$$

*for a CH robot i not visited by the UAV where $u_{i}$ is data forwarding strategy of CH robot i.*

The following example is given to better understand Definition 10.

**Figure 2:**
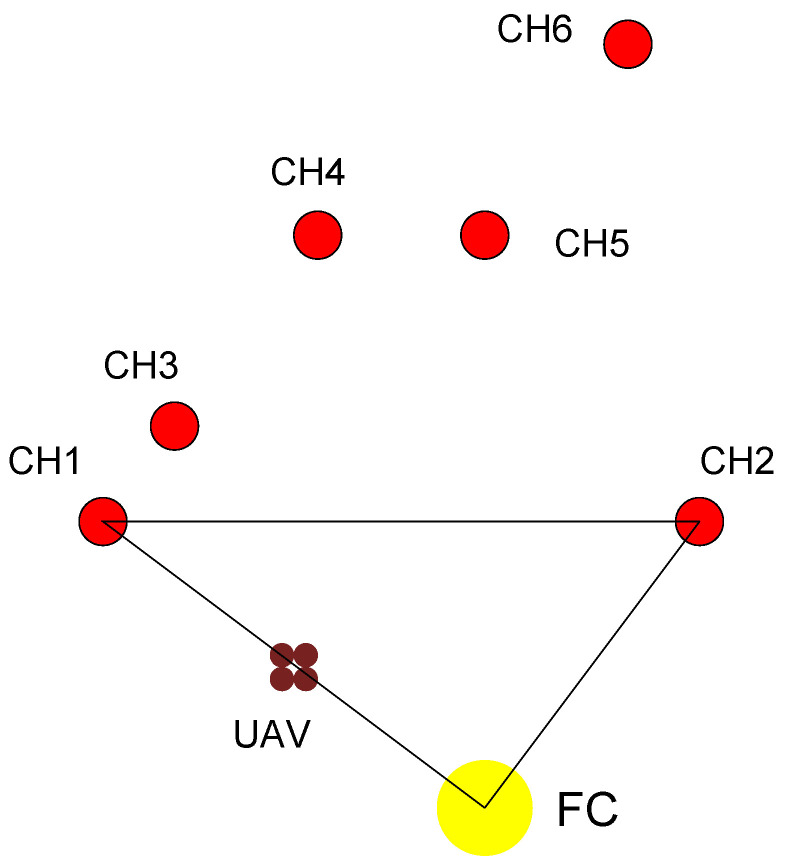
The whole multi-robot system consists of a fusion center (FC) where the UAV starts its route and six clusters of robots around FC. The red circles show the locations of the six cluster head (CH) robots in their robot cluster. *The UAV uses the strategy* $\pi_{0}$ *for the data collection*. With respect to this initial position of the UAV at $\xi_{0}=\left(0,0\right)$, the positions of the CH robots are $\left(\xi_{1},\xi_{2},\xi_{3},\xi_{4},\xi_{5},\xi_{6}\right)=\left((-16,12),(9,16),(-13,16),(-7,24),(0,24),(6,32)\right)$ units.

**Example** **1.** *Let us consider the multirobot system in Figure 2, where the UAV collects data from 6 CH robots by only visiting two of them under strategy $\pi_{0}$.*

*Under the strategy $\pi_{0}$, the UAV visits CH 1 and CH 2 (they are on the route) so $E_{1}^{\pi_{0}}\left(u_{1}\right)=E_{2}^{\pi_{0}}\left(u_{2}\right)=0$. Therefore, $\gamma_{1}^{\pi_{0}}=\gamma_{2}^{\pi_{0}}=0$.*

*If CH 3 choose CH 1 to forward its data under strategy $u_{3}$, then its energy consumption will be minimum, i.e.,*$\gamma_{3}^{\pi_{0}}=C_{CH}{∥\xi_{3}-\xi_{1}∥}^{2}=25$*for*$C_{CH}=1$*from Equation* (2). *Similarly, if CH 5 choose CH 2 to forward its data under strategy $u_{5}$, then its energy consumption will be minimum, i.e.,* $\gamma_{5}^{\pi_{0}}=C_{CH}{∥\xi_{5}-\xi_{2}∥}^{2}=225$ *for* $C_{CH}=1$ *from Equation* (2).

*If CH 4 forwards its data directly to CH 2, $E_{4}^{\pi_{0}}\left(u_{4}\right)=C_{CH}{∥\xi_{4}-\xi_{2}∥}^{2}=400$ for $C_{CH}=1$ from Equation (2). If CH 4 forwards its data directly to CH 1, $E_{4}^{\pi_{0}}\left(u_{4}\right)=C_{CH}{∥\xi_{4}-\xi_{1}∥}^{2}=225$ for $C_{CH}=1$ from Equation (2). If CH 4 forwards its data first to CH 5 then forwards it to CH 2, $E_{4}^{\pi_{0}}\left(u_{4}\right)=C_{CH}[∥\xi_{4}-\xi_{5}{∥}^{2}+∥\xi_{5}-\xi_{2}{∥}^{2}]=49+225=274$ for $C_{CH}=1$ from Equation (2). If CH 4 forwards its data first to CH 3 then forwards it to CH 1, $E_{4}^{\pi_{0}}\left(u_{4}\right)=C_{CH}[∥\xi_{4}-\xi_{3}{∥}^{2}+∥\xi_{3}-\xi_{1}{∥}^{2}]=100+25=125$ for $C_{CH}=1$ from Equation (2).* Hence, $\gamma_{4}^{\pi_{0}}=125$.

*If CH 6 forwards its data directly to CH 2, $E_{6}^{\pi_{0}}\left(u_{6}\right)=C_{CH}{∥\xi_{6}-\xi_{2}∥}^{2}=409$ for $C_{CH}=1$ from Equation (2). If CH 6 forwards its data first to CH 5 then to forwards CH 2, $E_{6}^{\pi_{0}}\left(u_{6}\right)=C_{CH}[∥\xi_{6}-\xi_{5}{∥}^{2}+∥\xi_{5}-\xi_{2}{∥}^{2}]=100+225=325$ for $C_{CH}=1$ from Equation (2). On the other hand, if CH 6 forwards its data directly to CH 1, $E_{6}^{\pi_{0}}\left(u_{6}\right)=C_{CH}{∥\xi_{6}-\xi_{1}∥}^{2}=884$ for $C_{CH}=1$ from Equation (2). If CH 6 forwards its data first to CH 5 then forwards it to CH 1, $E_{6}^{\pi_{0}}\left(u_{6}\right)=C_{CH}[∥\xi_{6}-\xi_{5}{∥}^{2}+∥\xi_{5}-\xi_{1}{∥}^{2}]=100+400=500$ for $C_{CH}=1$ from Equation (2). If CH 6 forwards its data first to CH 4 then forwards it to CH 1, $E_{6}^{\pi_{0}}\left(u_{6}\right)=C_{CH}[∥\xi_{6}-\xi_{4}{∥}^{2}+∥\xi_{4}-\xi_{1}{∥}^{2}]=233+225=458$ for $C_{CH}=1$ from Equation (2). If CH 6 forwards its data first to CH 5, then forwards it to CH 4 and finally then forwards it to CH 1, $E_{6}^{\pi_{0}}\left(u_{6}\right)=C_{CH}[∥\xi_{6}-\xi_{5}{∥}^{2}+∥\xi_{5}-\xi_{4}{∥}^{2}+∥\xi_{4}-\xi_{1}{∥}^{2}]=100+49+225=374$ for $C_{CH}=1$ from Equation (2). If CH 6 forwards its data first to CH 5, then forwards it to CH 4, after then forwards it to CH 3 and finally then forwards it to CH 1, $E_{6}^{\pi_{0}}\left(u_{6}\right)=C_{CH}[∥\xi_{6}-\xi_{5}{∥}^{2}+∥\xi_{5}-\xi_{4}{∥}^{2}+∥\xi_{4}-\xi_{3}{∥}^{2}+∥\xi_{3}-\xi_{1}{∥}^{2}]=100+49+100+25=274$ for $C_{CH}=1$ from Equation (2). Hence, $\gamma_{6}^{\pi_{0}}=274$.*

Following remarks will be useful for proposing the data forwarding strategy for CH robots.

**Remark** **4.** *In Example 1, notice that the best data forwarding strategy for CH 6 is to forward its data to CH 1 (the farther CH robot on the trajectory of the UAV) using CH 5, CH 4, or CH 3 instead of forwarding its data to CH 2 (the closer CH robot on the trajectory of the UAV).*

**Remark** **5.** *The data forwarding strategy of a CH robot i not visited by the UAV, $u_{i}$, depends only on the positions of the CH robots, both the CH robots on the trajectory of the UAV and the ones not on the trajectory of the UAV. Notice that $u_{i}$ does not depend on the order by which the UAV visit those CH robots. As an example, data forwarding strategies for a CH robot i is same for following two cases: (1) the UAV visits CH robots p, q, r, s in order, (2) the UAV visits CH robots q, p, s, r, in order (i∉{p,q,r,s}).*

By the motivation from Remark 5, we make the following definition to propose the data forwarding strategy for CH robots more precisely.

**Definition** **11** **(*K*-element combinations of the CH robots).** *Let $S_{a}^{K}$ be a K-element subset of the M-element set of all CH robots, i.e., $S_{a}^{K}\subseteq S$ and $|S_{K}^{a}|=K$ for 1≤a≤MK. Let $S^{K}\left(B\right)$ be the set of all feasible K-element combinations which can be visited by the UAV with battery capacity B.*

From Remark 4, the data forwarding strategy needs to consider the sum of squares of distances between CH robots instead of the sum of distances between CH robots. From Remark 5, the data forwarding strategy depends only on the positions of the CH robots not visited by the UAV and the ones visited by the UAV whereas the strategy does not depend on the order in which the UAV visits them. Thus, Remark 4 and Remark 5 provide us the motivation to propose optimal data forwarding strategy for a CH robot, Algorithm 1, by using Definition 11.
**Algorithm 1** Multi Shortest Path Data Forwarding Strategy (MSPDFS).**Initialization:** Assume that the UAV chose a *K*-element combination from *M* CH robots, $S_{a}^{K}$, to look for a trajectory to visit all CH robots in that combination. $|S_{a}^{K}|$ is cardinality of the set $S_{a}^{K}$. Given the combination $S_{a}^{K}$, data forwarding strategy is formed as follows.**Algorithm:****for**i=1:M**do**      # *Comment: If CH robot i is on the trajectory of the UAV*  **if** $i\in S_{a}^{K}$ **then**
           # *Comment: No need to forward data to another CH robot*           $u_{i}=\emptyset$ and $\gamma_{i}^{\pi }=0$  **else**
           # *Comment: Calculate shortest paths for all CH robot on the trajectory of the UAV*.    **for** $j=1:|S_{a}^{K}|$ **do**
           find shortest path (minimum energy path strategy for data forwarding) from the position of CH robot *i* to the position of CH robot $S_{a}^{K}\left(j\right)$.    **end for**
           Compare it with the shortest path to origin (initial position of the UAV) and choose the shorter one. find the optimal strategy $u_{i}^{*}=\arg\min_{u_{i}}E_{i}^{\pi }\left(u_{i}\right)$ by comparing the energy cost of the best strategy for each destination, $S_{a}^{K}\left(j\right)$.  **end if**
**end for****Output:** The optimal data forwarding strategy $u_{i}^{*}$ for each CH robot *i* given $S_{a}^{K}$

### 5.2. Optimal Strategy for the UAV

As it is exhibited in the previous section, if the UAV has sufficient battery capacity to visit all CH robots, then Problem 1 can be considered as a TSP problem and an optimal strategy can be obtained by common techniques in the literature. On the other hand, if the UAV has a battery capacity less than $B_{TSP}$, then considering Problem 1 as a TSP problem does not guarantee to obtain an optimal strategy.

In this case, we should consider not only the minimum energy consumption of the UAV but also the energy consumption of the CH robots not visited by the UAV (not on the trajectory of the UAV) and the data forwarding strategies for CH robots (They are investigated in the previous subsection.) This is shown as in the following proposition. Let us denote $\pi^{*}$ be the optimal strategy for Problem 1.

**Proposition** **1.** *For $B<B_{TSP}$, $\pi_{TSP}^{max}$ does not necessarily imply the optimality condition for the Problem 1, where $\pi_{TSP}^{max}$ be the strategy by which the TSP can visit visit the maximum number of CH robots.*

**Proof.**  Please see Appendix A. □

As the UAV with battery capacity $B<B_{TSP}$ does not have sufficient energy to visit all CH robots, it needs to desist from visiting some of the CH robots. The problem is to choose a subset of CH robots to desist from visiting them such that the total energy consumption of those unvisited CH robots will be minimum. The following remark will be useful in analyzing the desisting process.

**Remark** **6.** *To solve the problem for the UAV with battery capacity $B<B_{TSP}$, (Let us remind that from Definition 9, $B_{TSP}$ is the minimum energy required for the UAV to visit all CH robots.) our strategy will start at a point $B=B_{TSP}$, then the strategy will decrease the energy consumption of the UAV by desisting from visiting some CH robots. The decrease needs to be at least $B_{TSP}-B$.*

The following lemma is useful to search for a path after desisting from visiting a set of CH robots.

**Lemma** **1.** *Assume that the UAV with battery capacity $B_{1}$ can follow an optimal route such that it visit CH robot i, j, and k successively on the optimal route by which the UAV visits K<M CH robots ($B_{1}$ is the minimum energy required to follow that path). If the battery capacity of UAV decreased slightly such that $B_{2}<B_{1}$ and the UAV decided to desist from visiting CH robot j, simply visiting CH robot k just after visiting CH robot i (the direct line from CH robot i to CH robot j would not guarantee optimality in order to plan a route with the remaining K−1 CH robots except CH robot j).*

**Proof.**  Please see Appendix B. □

From Remark 5, The data forwarding strategy of a CH robot not visited by the UAV depends only on the positions of the CH robots on the trajectory of the UAV and the ones not on the trajectory of the UAV. The data forwarding strategies do not depend on the order in which the UAV visits them. This need to be considered for finding optimal data forwarding strategies. From Lemma 1, if the UAV desists to visit a CH robot *j*, instead of simply passing from the previous CH robot *i* to the next CH robot *k* according to the visiting order (*i*, *j*, *k* are the consecutive CH robots according to the order), the UAV needs to consider the path planning problem again as a TSP problem for finding the optimal path (trajectory). Therefore, the UAV can find the optimal path only considering path planning problem as a TSP problem for each combination of CH robots from Definition 11. Thus, Remark 5 and Lemma 1 provide us the motivation to propose Algorithm 2 by using Definition 11.
**Algorithm 2** Genetic Algorithm with Minimum Energy for Data Forwarding (GAMEDF).**Initialization:** Battery capacity of the UAV is insufficient for visiting all CH robots, i.e., $B<B_{TSP}$.**Algorithm:****for**K=(M−1):1**do**   # *Comment: Choose K of M CH robots and desist from visiting remaining CH robots.*   find all MK combinations of the CH robots.   # *Comment: For each of the MK combinations of CH robots.*   **for** a=1:MK **do**
      # *Comment: Check whether the UAV can find a route to visit all CH robots in the combination $S_{a}^{K}$.*      **if** $\min_{\pi }E_{UAV}^{\pi }\leq B$ for $S_{a}^{K}$ **then**         # *Comment: $S_{a}^{K}$ is a feasible K-element combination, i.e., $S_{a}^{K}\in S^{K}\left(B\right)$. find the minimum energy consumption of the CH robots in $S-S_{a}^{K}$.*         Use Algorithm 1 for each CH robot $i\in S-S_{a}^{K}$. find $\sum_{i\in S-S_{a}^{K}}\gamma_{i}^{\pi }$     **end if**
  **end for**
  # *Comment: If there exists at least a feasible K-element combination, find the K-element combination $S_{a}^{K}$ by which energy consumption of CH robots in $S-S_{a}^{K}$ will be minimum.*
  **if** $S^{K}\left(B\right)\neq \emptyset$ **then**
      find $\min_{S_{a}^{K}\in S^{K}\left(B\right)}\sum_{i\in S-S_{a}^{K}}\gamma_{i}^{\pi }$
  **end if**
**end for**    # *Comment: find the combination $S_{a}^{K}$ by which the minimum energy consumption of the CH robots in $S-S_{a}^{K}$ will be minimum (In this step, all K values are considered so this is the step to find the minimum of minimum).*    find $\min_{K}\left[\min_{S_{a}^{K}\in S^{K}\left(B\right)}\sum_{i\in S-S_{a}^{K}}\gamma_{i}^{\pi }\right]$.    # *Comment: As output, provide the result of K and the combination $S_{a}^{K}$ as the solution.***Output:**$\left(K,S_{a}^{K}\right)=\arg\min\sum_{i\in S-S_{a}^{K}}\gamma_{i}^{\pi }$

**Theorem** **1.** *Algorithm 2 is optimal for Problem 1.*

**Proof.**  From Definition 8, Problem 1 can be converted into the following problem

(10) $$\begin{array}{cccc} \; & \min_{\pi \subseteq P}\min_{u_{i}}\; & \; & \sum_{i=1}^{M}\left[E_{i}^{\pi }\left(u_{i}\right)I_{\left\{i\in S-S_{a}^{K}\right\}}\right]\\ \; & s.t.\; & \; & E_{UAV}^{\pi }\leq B \end{array}$$

where *P* is the route of the UAV to visit the CH robots. $S_{a}^{K}$ be a *K*-element subset of the *M*-element set of all CH robots, i.e., $S_{a}^{K}\subseteq S$ and $|S_{K}^{a}|=K$ for 1≤a≤MK.

From Definition 10, the problem in (10) can be converted into the following problem

(11) $$\begin{array}{cccc} \; & \min_{\pi \subseteq P}\; & \; & \sum_{i=1}^{M}\left[\gamma_{i}^{\pi }I_{\left\{i\in S-S_{a}^{K}\right\}}\right]\\ \; & s.t.\; & \; & E_{UAV}^{\pi }\leq B \end{array}$$

From Remark 6, the UAV with battery capacity $B<B_{TSP}$ cannot visit all *M* CH robots; therefore, the UAV need to desist from visiting some of the CH robots.

From Remark 5, the strategy of a CH robot *i* not visited by the UAV, $u_{i}$, depends only on the positions of the CH robots. $u_{i}$ does not depend on the route on which the UAV visit the CH robots. Therefore, the total energy consumption of the CH robots depends on the combination by which the UAV determines the CH robots to visit.

If $\min_{\pi }E_{UAV}^{\pi }>B$ for $S_{a}^{K}$, then the combination $S_{a}^{K}$ is such an infeasible combination that the UAV with battery capacity *B* cannot visit all the CH robots in that combination $S_{a}^{K}$. Therefore, it is unnecessary to find $\sum_{i\in S-S_{a}^{K}}\gamma_{i}^{\pi }$ for $S_{a}^{K}$ such that $\min_{\pi }E_{UAV}^{\pi }>B$ for $S_{a}^{K}$.

Algorithm 2 finds all $\sum_{i\in S-S_{a}^{K}}\gamma_{i}^{\pi }$ for $S_{a}^{K}\in S^{K}\left(B\right)$ (remind that $\min_{\pi }E_{UAV}^{\pi }\leq B$ for $S_{a}^{K}\in S^{K}\left(B\right)$ from Definition 11) and then takes the minimum of $\sum_{i\in S-S_{a}^{K}}\gamma_{i}^{\pi }$ values.

From Proposition 1, the following inequality cannot be guaranteed

(12) $$\min_{S_{a}^{K}\in S^{K}\left(B\right)}\sum_{i\in S-S_{a}^{K}}\gamma_{i}^{\pi }\leq \min_{S_{a}^{K-1}\in S^{K-1}\left(B\right)}\sum_{i\in S-S_{a}^{K-1}}\gamma_{i}^{\pi }$$

Therefore, the algorithm consider all *K* values such that 1≤K≤M−1 for optimal solution. In other words, an algorithm need to find $\min_{K}\left[\min_{S_{a}^{K}\in S^{K}\left(B\right)}\sum_{i\in S-S_{a}^{K}}\gamma_{i}^{\pi }\right]$ to guarantee optimality.

Thus, Algorithm 2 guarantees optimality for the problem in 11. Hence, Algorithm 2 guarantees the optimality for Problem 1. □

## 6. Numerical Results

In this section, we will evaluate the performance of the strategies for varying battery capacities and varying number of CH robots. We consider three scenarios with varying number of CH robots, namely 5-CH robot scenario, 7-CH robot scenario, and 10-CH robot scenario. In each scenario, the locations of CH robots are randomly generated. For different battery capacities, the performance of different strategies are investigated.

In these scenarios, we observe that the path length for the UAV to visit all CH robots is less than 50 units. This means that the battery capacity of the UAV is sufficient to visit all CH robots is less than $50\times C_{UAV}$ (remind that $C_{UAV}$ is the energy consumption of the UAV per unit distance travel). In these scenarios, we evaluate the performances of two strategies, namely, our optimal (GAMEDF) strategy and UAV-oriented strategy in [40]. Please notice that we investigates all decisions taken by both strategies for each battery level varying from $B=50\times C_{UAV}$ to $B=5\times C_{UAV}$ in each of 5-CH, 7-CH and 10-CH robot scenarios. We also calculate the resultant energy consumption of the UAV and CH robots.

### 6.1. 5-CH Case

**Figure 3:**
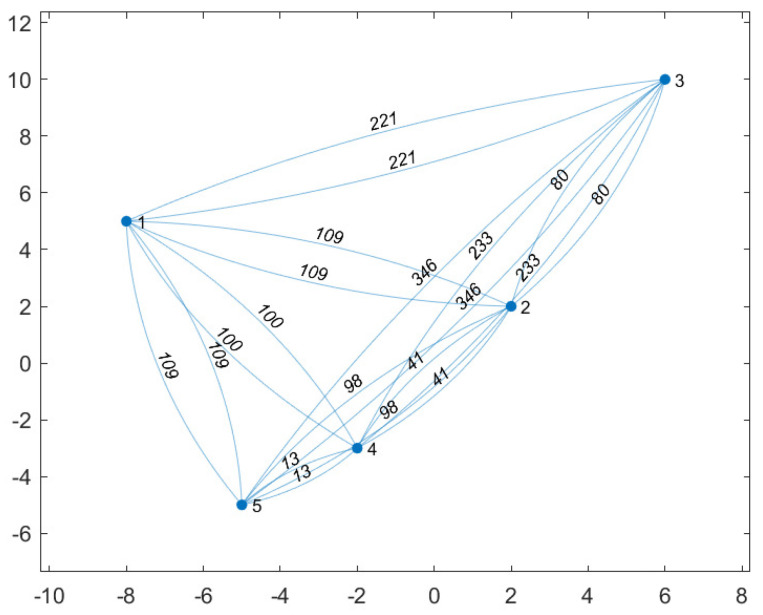
Nodes show that the locations (positions) of the five CH robots. The weight of a link shows the square of distance between the two nodes connected via that link.

Figure 3 shows the locations of the five CH robots and the weights of the links between them. With respect to this initial position of the UAV (0,0), the positions of the CH robots are $\left(\xi_{1},\xi_{2},\xi_{3},\xi_{4},\xi_{5}\right)=\left((-8,5),(2,2),(6,10),(-2,-3),(-5,-5)\right)m$. In the configuration in Figure 3, the total energy consumption is $296\times C_{CH}$ if all CH robots send their data directly to the FC at $\xi_{0}=\left(0,0\right)$ (the UAV visit no CH robot).

By applying the UAV-oriented strategy, the UAV travels only to CH robot 2 ($\xi_{2}=\left(2,2\right)$) and collects all data of the CH robots from there if the UAV has sufficient battery capacity (to travel there and return back to the FC), which is

(13) $$\begin{array}{ccc} B & = & 2\times ∥\xi_{2}-\xi_{0}∥\\ = & 2\times \sqrt{{\left(2-0\right)}^{2}+{\left(2-0\right)}^{2}}\times C_{UAV}\approx 5.66\times C_{UAV}<10\times C_{UAV}, \end{array}$$

by which the UAV can apply the UAV-Oriented strategy which results in the total energy consumption of the CH robots as follows

(14) $$\begin{array}{ccc} E_{ACH}^{\pi^{UAV-Oriented}}\left(u\right) & = & ∥\xi_{1}-\xi_{2}{∥}^{2}+∥\xi_{3}-\xi_{2}{∥}^{2}+∥\xi_{4}-\xi_{2}{∥}^{2}+{∥\xi_{5}-\xi_{2}∥}^{2}\\ = & \left(109+80+41+98\right)\times C_{CH}=328\times C_{CH}>296\times C_{CH}, \end{array}$$

which yields an interesting result that the UAV-oriented strategy causes more energy consumption of CH robots than no strategy (the UAV visits no CH robots) in the configuration in Figure 3. If B=5, then the UAV-oriented strategy is not applicable because the UAV cannot travel to CH robot 2 and none of the other CH robots are closer to the origin than CH robot 2.

**Figure 4:**
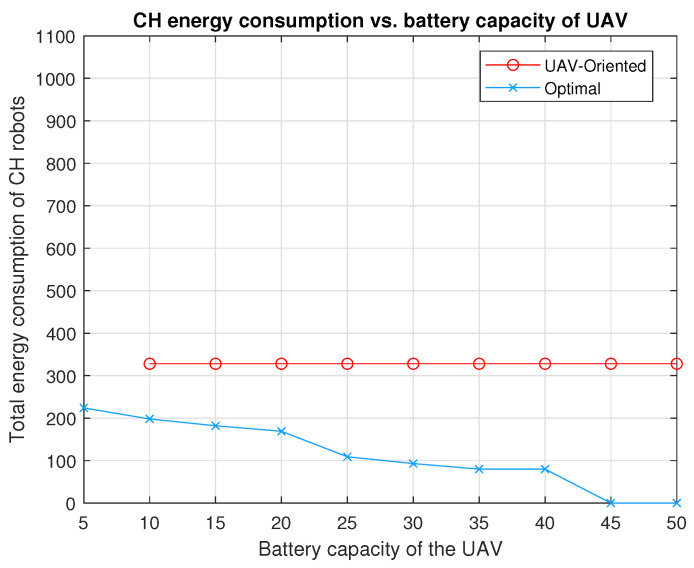
The total energy consumption of the five CH robots in Figure 3 under the strategies, UAV-oriented and optimal for varying battery capacities of the UAV from $B=5\times C_{UAV}$ to $B=50\times C_{UAV}$.

The performance of optimal strategy is investigated for battery capacities varying from B=5 to B=50 in the configuration in Figure 3. In Figure 4, by applying optimal strategy, the UAV with $B=45\times C_{UAV}$ or $B=50\times C_{UAV}$ can make the CH robots consume no energy for forwarding data ($E_{UAV}^{\pi^{*}}\left(u\right)=0$). Notice that if the problem is considered as a TSP problem for the configuration in Figure 3, the energy required for the UAV to visit all CH robots is

(15) $$\begin{array}{ccc} E_{UAV}^{\pi^{*}}\left(u\right) & = & ∥\xi_{0}-\xi_{2}∥+∥\xi_{2}-\xi_{3}∥+∥\xi_{3}-\xi_{1}∥+∥\xi_{1}-\xi_{5}∥+∥\xi_{5}-\xi_{4}∥+∥\xi_{4}-\xi_{0}∥\\ = & \left(\sqrt{8}+\sqrt{80}+\sqrt{221}+\sqrt{109}+\sqrt{13}+\sqrt{13}\right)\times C_{UAV}\approx 44.29\times C_{UAV} \end{array}$$

which yields that the UAV with B=44.29 need to desist from visiting at least one CH robot. The UAV with B=35 or $B=40\times C_{UAV}$ desists from visiting CH robot 3 which results in $80\times C_{CH}$ total energy consumption of CH robots. Thus, the UAV consumes

(16) $$\begin{array}{ccc} E_{UAV}^{\pi^{*}}\left(u\right) & = & ∥\xi_{0}-\xi_{2}∥+∥\xi_{2}-\xi_{1}∥+∥\xi_{1}-\xi_{5}∥+∥\xi_{5}-\xi_{4}∥+∥\xi_{4}-\xi_{0}∥\\ = & \left(\sqrt{8}+\sqrt{109}+\sqrt{109}+\sqrt{13}+\sqrt{13}\right)\times C_{UAV}\approx 30.92\times C_{UAV} \end{array}$$

The UAV with $B=30\times C_{UAV}$ desists from visiting CH robot 3 and CH robot 5, which results in $80+13=93\times C_{CH}$ total energy consumption of CH robots. Thus, the UAV consumes

(17) $$\begin{array}{ccc} E_{UAV}^{\pi^{*}}\left(u\right) & = & ∥\xi_{0}-\xi_{2}∥+∥\xi_{2}-\xi_{1}∥+∥\xi_{1}-\xi_{4}∥+∥\xi_{4}-\xi_{0}∥\\ = & \left(\sqrt{8}+\sqrt{109}+\sqrt{100}+\sqrt{13}\right)\times C_{UAV}\approx 26.87\times C_{UAV} \end{array}$$

The UAV with $B=25\times C_{UAV}$ desists from visiting CH robot 3, CH robot 2, and CH robot 5 which results in $\left(80+8\right)+8+13=109\times C_{CH}$ total energy consumption of CH robots (CH 3 robot sends its data to CH robot 2 to forward it to the UAV at FC). Thus, the UAV consumes

(18) $$\begin{array}{ccc} E_{UAV}^{\pi^{*}}\left(u\right) & = & ∥\xi_{0}-\xi_{1}∥+∥\xi_{1}-\xi_{4}∥+∥\xi_{4}-\xi_{0}∥\\ = & \left(\sqrt{89}+\sqrt{100}+\sqrt{13}\right)\times C_{UAV}\approx 23.04\times C_{UAV} \end{array}$$

The UAV with $B=20\times C_{UAV}$ desists from visiting CH robot 3 and CH robot 1 which results in $80+89=169\times C_{CH}$ total energy consumption of CH robots. Thus, the UAV consumes

(19) $$\begin{array}{ccc} E_{UAV}^{\pi^{*}}\left(u\right) & = & ∥\xi_{0}-\xi_{2}∥+∥\xi_{2}-\xi_{5}∥+∥\xi_{5}-\xi_{4}∥+∥\xi_{4}-\xi_{0}∥\\ = & \left(\sqrt{8}+\sqrt{98}+\sqrt{13}+\sqrt{13}\right)\times C_{UAV}\approx 19.94\times C_{UAV} \end{array}$$

The UAV with $B=15\times C_{UAV}$ desists from visiting CH robot 3, CH robot 5, and CH robot 1 which results in $80+13+89=182\times C_{CH}$ total energy consumption of CH robots. Thus, the UAV consumes

(20) $$\begin{array}{ccc} E_{UAV}^{\pi^{*}}\left(u\right) & = & ∥\xi_{0}-\xi_{2}∥+∥\xi_{2}-\xi_{4}∥+∥\xi_{4}-\xi_{0}∥\\ = & \left(\sqrt{8}+\sqrt{41}+\sqrt{13}\right)\times C_{UAV}\approx 12.84\times C_{UAV} \end{array}$$

The UAV with $B=10\times C_{UAV}$ desists from visiting CH robot 3, CH robot 2, CH robot 5, and CH robot 1 which results in $\left(80+8\right)+8+13+89=198\times C_{CH}$ total energy consumption of CH robots. Thus,

(21) $$\begin{array}{ccc} E_{UAV}^{\pi^{*}}\left(u\right) & = & ∥\xi_{0}-\xi_{4}∥+∥\xi_{4}-\xi_{0}∥\\ = & \left(\sqrt{13}+\sqrt{13}\right)\times C_{UAV}\approx 7.21\times C_{UAV} \end{array}$$

The UAV with $B=5\times C_{UAV}$ cannot travel to any CH robots which results in $\left(80+8\right)+8+\left(13+13\right)+13+89=224\times C_{CH}$ total energy consumption of CH robots (CH 3 robot and CH robot 5 send its data to CH robot 2 and CH robot 4, respectively, in order to forward it to the UAV at the origin). Even without visiting any CH robot, optimal strategy performs better than no strategy and the UAV-oriented strategy.

Table 2 summarizes indices of the nonvisited CH robots which the strategies decide to desist from visiting in the configuration of Figure 3. Similarly, Table 3 summarizes total energy consumption of the nonvisited CH robots which the strategies decides to desist from visiting. From these tables, it can be observed how UAV decides to desist from visiting a subset of CH robots depending on its battery capacity in the configuration of Figure 3. Furthermore, the total energy consumption of the nonvisited CH robots depends on the desisting decisions taken by UAV and so the battery capacity of the UAV. Besides these, the total energy consumption of the nonvisited CH robots also depends on the network topology, the locations of all CH robots.

We compare the performances of UAV-Oriented strategy and our two-stage optimal strategy for battery capacities varying from B=5 to B=50 in the configuration in Figure 3. From Figure 4, the following observations can be made. Applying the UAV-oriented strategy results in $328\times C_{CH}$ for battery capacities from B=10 to B=50, it is not applicable for B=5 which is insufficient battery capacity for the UAV to travel any CH robot and turn back to the FC. This strategy performs worse than no strategy by which the UAV standing on the FC collects data from all CH robots (no strategy results in $296\times C_{CH}<328\times C_{CH}$). On the other hand, the two-stage optimal strategy achieves zero energy consumption of all CH robots for B=45 and B=50. Optimal strategy results in $80\times C_{CH}$ total energy consumption, only one fourth of that by the UAV-oriented strategy for B=35 and B=40. Optimal strategy results in $93\times C_{CH}$ and $109\times C_{CH}$ total energy consumption, less than one third of that by the UAV-oriented strategy, for B=30 and B=25, respectively. For B=20, B=15 and B=10, optimal strategy results in $169\times C_{CH}$, $182\times C_{CH}$ and $198\times C_{CH}$ total energy consumption, respectively, still less than 61% of that by the UAV-oriented strategy. Even for B=5, optimal strategy results in $224\times C_{CH}$ total energy consumption, still less than 76% of that by no strategy. Remind that UAV-Oriented strategy is not applicable for B=5.

### 6.2. 7-CH Case

**Figure 5:**
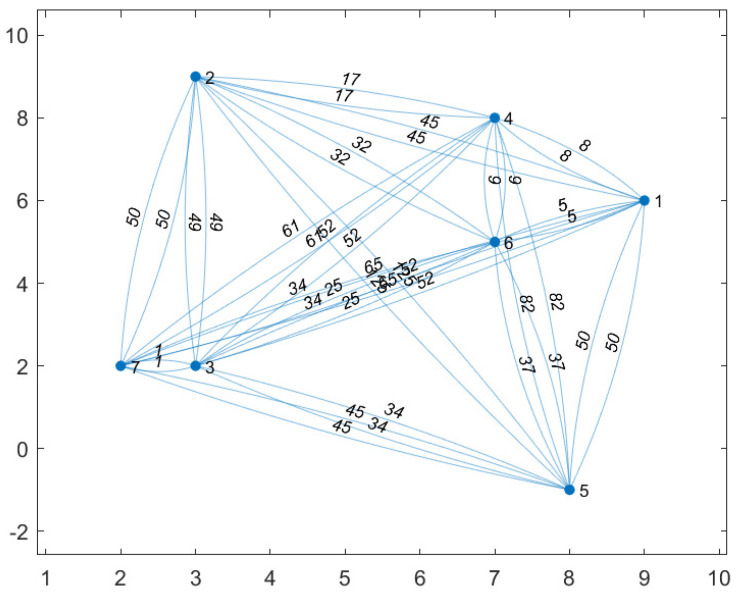
Nodes show that the locations of the seven CH robots. The weight of a link shows the square of distance between the two nodes connected via that link.

Figure 5 shows the locations of the seven CH robots and the weights of the links between them. With respect to this initial position of the UAV (0,0), the positions of the CH robots are $\left(\xi_{1},\xi_{2},\xi_{3},\xi_{4},\xi_{5},\xi_{6},\xi_{7}\right)=\left((9,6),(3,9),(3,2),(7,8),(8,-1),(7,5),(2,2)\right)m$. In the configuration in Figure 5, the total energy consumption is $480\times C_{CH}$ if all CH robots send their data directly to the FC at $\xi_{0}=\left(0,0\right)$ (the UAV visit no CH robot).

By applying the UAV-oriented strategy, the UAV travels only to CH robot 6 ($\xi_{6}=\left(7,5\right)$) and collect all data of the CH robots from there if the UAV has sufficient battery capacity (to travel there and return back to the FC), which is

(22) $$\begin{array}{ccc} B & = & 2\times ∥\xi_{6}-\xi_{0}∥\\ = & 2\times \sqrt{{\left(7-0\right)}^{2}+{\left(5-0\right)}^{2}}\approx 17.2\times C_{UAV}<20\times C_{UAV}, \end{array}$$

by which the UAV can apply the UAV-oriented strategy which results in the total energy consumption of the CH robots as follows

(23) $$\begin{array}{c} E_{ACH}^{\pi^{UAV-Oriented}}\left(u\right)=∥\xi_{1}-\xi_{6}{∥}^{2}+∥\xi_{2}-\xi_{6}{∥}^{2}+∥\xi_{3}-\xi_{6}{∥}^{2}+∥\xi_{4}-\xi_{6}{∥}^{2}+∥\xi_{5}-\xi_{6}{∥}^{2}+{∥\xi_{7}-\xi_{6}∥}^{2}\\ \;\;\;\;\;\;\;\;\;\;\;\;\;\;\;\;\;\;\;\;\;\;=\left(5+32+25+9+37+34\right)\times C_{CH}=142\times C_{CH} \end{array}$$

If B=10 or B=15, the UAV can travel to CH robot 3 which is closer to the origin than CH robot 6. Similar to Equation (23), $E_{ACH}^{\pi^{UAV-Oriented}}\left(u\right)=213\times C_{CH}$ However, if B=5, then the UAV-oriented strategy is not applicable because the UAV cannot travel to any CH robot.

**Figure 6:**
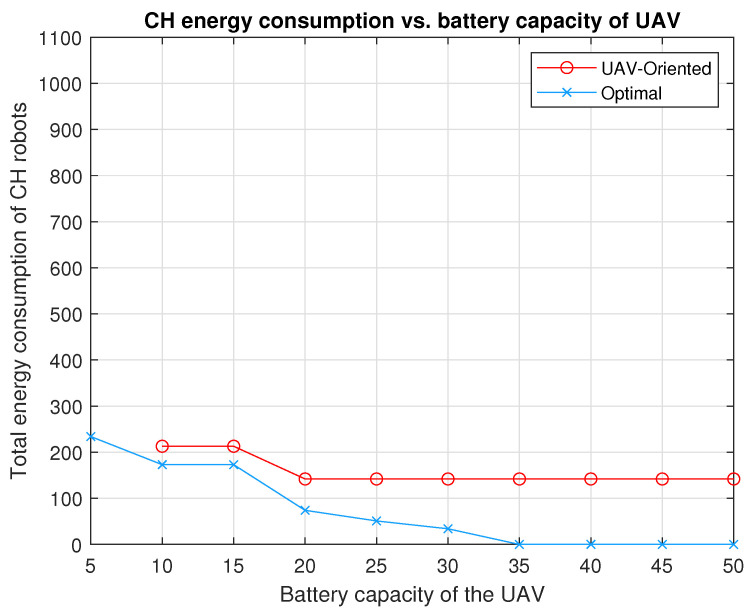
The total energy consumption of the seven CH robots in Figure 5 under the strategies, UAV-oriented and optimal for varying battery capacities of the UAV from $B=5\times C_{UAV}$ to $B=50\times C_{UAV}$.

The performance of optimal strategy is investigated for battery capacities varying from B=5 to B=50 in the configuration in Figure 5. In Figure 6, by applying optimal strategy, the UAV with $B=35\times C_{UAV}$ or $B=40\times C_{UAV}$ or $B=45\times C_{UAV}$ or $B=50\times C_{UAV}$ can make the CH robots consume no energy for forwarding data ($E_{UAV}^{\pi^{*}}\left(u\right)=0$). Notice that if the problem is considered as a TSP problem for the configuration in Figure 5, the energy required for the UAV to visit all CH robots is

(24) $$\begin{array}{ccc} E_{UAV}^{\pi^{*}}\left(u\right) & = & ∥\xi_{0}-\xi_{2}∥+∥\xi_{2}-\xi_{4}∥+∥\xi_{4}-\xi_{1}∥+∥\xi_{1}-\xi_{6}∥+∥\xi_{6}-\xi_{5}∥+∥\xi_{5}-\xi_{3}∥\\ + & ∥\xi_{3}-\xi_{7}∥+∥\xi_{7}-\xi_{0}∥\\ = & \left(\sqrt{90}+\sqrt{17}+\sqrt{8}+\sqrt{5}+\sqrt{37}+\sqrt{34}+1+\sqrt{8}\right)\times C_{UAV}\approx 34.42\times C_{UAV} \end{array}$$

which yields that the UAV with $B<34.42\times C_{UAV}$ needs to desist from visiting at least one CH robot. The UAV with $B=30\times C_{UAV}$ desists from visiting CH robot 5 which results in $34\times C_{CH}$ total energy consumption of CH robots. Thus, the UAV consumes

(25) $$\begin{array}{c} E_{UAV}^{\pi^{*}}\left(u\right)=∥\xi_{0}-\xi_{2}∥+∥\xi_{2}-\xi_{4}∥+∥\xi_{4}-\xi_{1}∥+∥\xi_{1}-\xi_{6}∥+∥\xi_{6}-\xi_{3}∥+∥\xi_{3}-\xi_{7}∥+∥\xi_{7}-\xi_{0}∥\\ \;\;\;\;\;\;\;\;\;\;\;\;\;=\left(\sqrt{90}+\sqrt{17}+\sqrt{8}+\sqrt{5}+5+1+\sqrt{8}\right)\times C_{UAV}\approx 27.50\times C_{UAV} \end{array}$$

The UAV with $B=25\times C_{UAV}$ desists from visiting CH robot 2 and CH robot 5 which results in $17+34=51\times C_{CH}$ total energy consumption of CH robots. Thus, the UAV consumes

(26) $$\begin{array}{ccc} E_{UAV}^{\pi^{*}}\left(u\right) & = & ∥\xi_{0}-\xi_{4}∥+∥\xi_{4}-\xi_{1}∥+∥\xi_{1}-\xi_{6}∥+∥\xi_{6}-\xi_{3}∥+∥\xi_{3}-\xi_{7}∥+∥\xi_{7}-\xi_{0}∥\\ = & \left(\sqrt{113}+\sqrt{8}+\sqrt{5}+5+1+\sqrt{8}\right)\times C_{UAV}\approx 24.52\times C_{UAV} \end{array}$$

The UAV with $B=20\times C_{UAV}$ desists from visiting CH robot 1, CH robot 2, CH robot 4, and CH robot 5 which results in $5+\left(17+9\right)+9+34=74\times C_{CH}$ total energy consumption of CH robots. Thus, the UAV consumes

(27) $$\begin{array}{ccc} E_{UAV}^{\pi^{*}}\left(u\right) & = & ∥\xi_{0}-\xi_{6}∥+∥\xi_{6}-\xi_{3}∥+∥\xi_{3}-\xi_{7}∥+∥\xi_{7}-\xi_{0}∥\\ = & \left(\sqrt{74}+5+1+\sqrt{8}\right)\times C_{UAV}\approx 17.43\times C_{UAV} \end{array}$$

The UAV with $B=10\times C_{UAV}$ or $B=15\times C_{UAV}$ desists from visiting CH robot 1, CH robot 2, CH robot 4, CH robot 5, and CH robot 6 which results in $\left(5+25\right)+50+\left(9+25\right)+34+25=173\times C_{CH}$ total energy consumption of CH robots. Thus, the UAV consumes

(28) $$\begin{array}{ccc} E_{UAV}^{\pi^{*}}\left(u\right) & = & ∥\xi_{0}-\xi_{3}∥+∥\xi_{3}-\xi_{7}∥+∥\xi_{7}-\xi_{0}∥\\ = & \left(\sqrt{13}+1+\sqrt{8}\right)\times C_{UAV}\approx 7.43\times C_{UAV} \end{array}$$

The UAV with $B=5\times C_{UAV}$ cannot travel to any CH robots which results in $\left(5+25+1+8\right)+\left(50+8\right)+\left(1+8\right)+\left(9+25+1+8\right)+\left(34+1+8\right)+\left(25+1+8\right)+8=234\times C_{CH}$ total energy consumption of CH robots (All CH robots send their data directly or indirectly to CH robot 7 to forward it to the UAV at FC). Even visiting no CH robot, optimal strategy performs better than no strategy.

Table 4 summarizes indices of the nonvisited CH robots which the strategies decide to desist from visiting in the configuration of Figure 5. Similarly, Table 5 summarizes total energy consumption of the nonvisited CH robots which the strategies decides to desist from visiting. From these tables, it can be observed how UAV decides to desist from visiting a subset of CH robots depending on its battery capacity in the configuration of Figure 5. Furthermore, the total energy consumption of the nonvisited CH robots depends on the desisting decisions taken by UAV and so the battery capacity of the UAV. Besides these, the total energy consumption of the nonvisited CH robots also depends on the network topology, the locations of all CH robots.

We compare the performances of the UAV-oriented strategy and our two-stage optimal strategy for battery capacities varying from B=5 to B=50 in the configuration in Figure 5. From Figure 6, the following observations can be made. Applying the UAV-oriented strategy results in $142\times C_{CH}$ and $213\times C_{CH}$ for battery capacities B=20,25,30,35,40,45,50 and B=10,15, respectively. It is not applicable for B=5 which is insufficient battery capacity for the UAV to travel any CH robot and turn back to the FC. On the other hand, the two-stage optimal strategy achieves zero energy consumption of all CH robots for B=35, B=40, B=45 and B=50. Optimal strategy results in $34\times C_{CH}$ total energy consumption, only one fourth of that by the UAV-oriented strategy for B=30. Optimal strategy results in $51\times C_{CH}$, less than 36% of that by the UAV-oriented strategy for B=25. For B=20, optimal strategy results in $51\times C_{CH}$, nearly half of that by the UAV-oriented strategy. For B=10 and B=15, optimal strategy results in $173\times C_{CH}$ total energy consumption, nearly 81% of that by the UAV-oriented strategy. Even for B=5, optimal strategy results in $234\times C_{CH}$ total energy consumption, still less than half of that by no strategy. Remember that the UAV-oriented strategy is not applicable for B=5.

### 6.3. 10-CH Case

**Figure 7:**
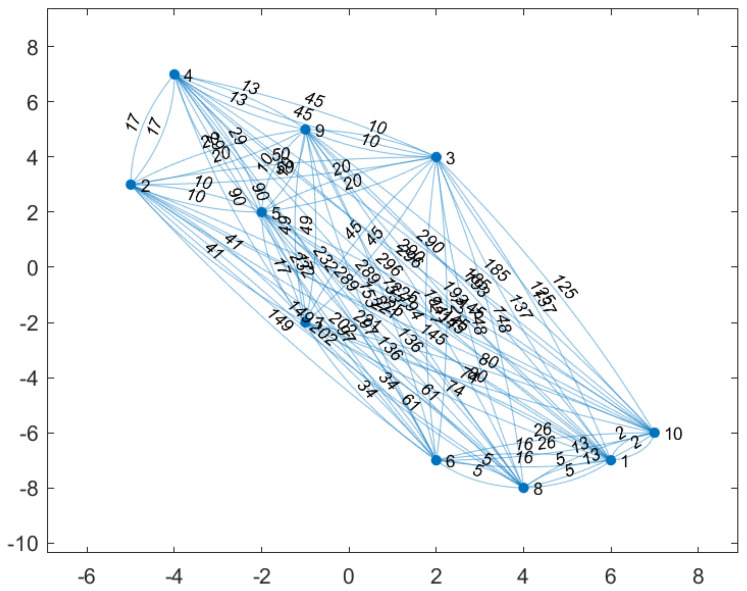
Nodes show that the locations of the 10 CH robots. The weight of a link shows the square of distance between the two nodes connected via that link.

Figure 7 shows the locations of the 10 CH robots and the weights of the links between them. With respect to this initial position of the UAV (0,0), the positions of the CH robots are $\left(\xi_{1},\xi_{2},\xi_{3},\xi_{4},\xi_{5},\xi_{6},\xi_{7},\xi_{8},\xi_{9},\xi_{10}\right)=(\left(6,-7\right),\left(-5,3\right),\left(2,4\right),\left(-4,7\right),\left(-2,2\right),\left(2,-7\right),\left(-1,-2\right)$, (4, −8), (−1, 5), (7,−6))m. In the configuration in Figure 7, the total energy consumption is $461\times C_{CH}$ if all CH robots send their data directly to the FC at $\xi_{0}=\left(0,0\right)$ (the UAV visit no CH robot).

By applying the UAV-oriented strategy, the UAV travels only to CH robot 7 ($\xi_{7}=\left(-1,-2\right)$) and collect all data of the CH robots from there if the UAV has sufficient battery capacity (to travel there and return back to the FC), which is

(29) $$\begin{array}{ccc} B & = & 2\times ∥\xi_{7}-\xi_{0}∥\\ = & 2\times \sqrt{{\left(-1-0\right)}^{2}+{\left(2-0\right)}^{2}}\times C_{UAV}\approx 4.47\times C_{UAV}<5\times C_{UAV}, \end{array}$$

by which the UAV can apply the UAV-oriented strategy which results in the total energy consumption of the CH robots as follows

(30) $$\begin{array}{ccc} E_{ACH}^{\pi^{UAV-Oriented}}\left(u\right) & = & ∥\xi_{1}-\xi_{7}{∥}^{2}+∥\xi_{2}-\xi_{7}{∥}^{2}+∥\xi_{3}-\xi_{7}{∥}^{2}+∥\xi_{4}-\xi_{7}{∥}^{2}+{∥\xi_{5}-\xi_{7}∥}^{2}\\ + & ∥\xi_{6}-\xi_{7}{∥}^{2}+∥\xi_{8}-\xi_{7}{∥}^{2}+∥\xi_{9}-\xi_{7}{∥}^{2}+{∥\xi_{10}-\xi_{7}∥}^{2}\\ = & \left(74+41+45+90+17+34+61+49+80\right)\times C_{CH}=491\times C_{CH} \end{array}$$

which yields an interesting result that the UAV-oriented strategy causes more energy consumption of CH robots than no strategy (the UAV visits no CH robots) in the configuration in Figure 7.

**Figure 8:**
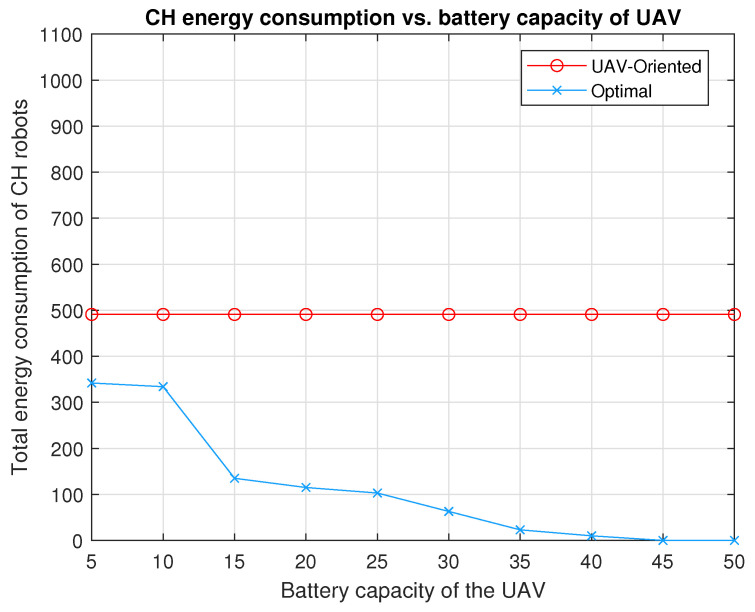
The total energy consumption of the 10 CH robots in Figure 7 under the strategies, UAV-oriented and optimal for varying battery capacities of the UAV from $B=5\times C_{UAV}$ to $B=50\times C_{UAV}$.

The performance of optimal strategy is investigated for battery capacities varying from B=5 to B=50 in the configuration in Figure 7. In Figure 8, by applying optimal strategy, the UAV with $B=45\times C_{UAV}$ or $B=50\times C_{UAV}$ can make the CH robots consume no energy for forwarding data ($E_{UAV}^{\pi^{*}}\left(u\right)=0$). Notice that if the problem is considered as a TSP problem for the configuration in Figure 7, the energy required for the UAV to visit all CH robots is

(31) $$\begin{array}{ccc} E_{UAV}^{\pi^{*}}\left(u\right) & = & ∥\xi_{0}-\xi_{5}∥+∥\xi_{5}-\xi_{2}∥+∥\xi_{2}-\xi_{4}∥+∥\xi_{4}-\xi_{9}∥+∥\xi_{9}-\xi_{3}∥+∥\xi_{3}-\xi_{10}∥\\ + & ∥\xi_{10}-\xi_{1}∥+∥\xi_{1}-\xi_{8}∥+∥\xi_{8}-\xi_{6}∥+∥\xi_{6}-\xi_{7}∥+∥\xi_{7}-\xi_{0}∥\\ = & \left(\sqrt{8}+\sqrt{10}+\sqrt{17}+\sqrt{13}+\sqrt{10}+\sqrt{125}+\sqrt{2}+\sqrt{5}+\sqrt{5}+\sqrt{34}+\sqrt{5}\right)\times C_{UAV}\\ \approx & 42.02\times C_{UAV} \end{array}$$

which yields that the UAV with $B<42.02\times C_{UAV}$ needs to desist from visiting at least one CH robot. The UAV with $B=40\times C_{UAV}$ desists from visiting CH robot 2 which results in $10\times C_{CH}$ total energy consumption of CH robots. Thus, the UAV consumes

(32) $$\begin{array}{ccc} E_{UAV}^{\pi^{*}}\left(u\right) & = & ∥\xi_{0}-\xi_{5}∥+∥\xi_{5}-\xi_{4}∥+∥\xi_{4}-\xi_{9}∥+∥\xi_{9}-\xi_{3}∥+∥\xi_{3}-\xi_{10}∥+∥\xi_{10}-\xi_{1}∥\\ + & ∥\xi_{1}-\xi_{8}∥+∥\xi_{8}-\xi_{6}∥+∥\xi_{6}-\xi_{7}∥+∥\xi_{7}-\xi_{0}∥\\ = & \left(\sqrt{8}+\sqrt{20}+\sqrt{13}+\sqrt{10}+\sqrt{125}+\sqrt{2}+\sqrt{5}+\sqrt{5}+\sqrt{34}+\sqrt{5}\right)\times C_{UAV}\\ \approx & 39.21\times C_{UAV} \end{array}$$

The UAV with $B=35\times C_{UAV}$ desists from visiting CH robot 2 and CH robot 4 which results in $10+13=23\times C_{CH}$ total energy consumption of CH robots. Thus, the UAV consumes

(33) $$\begin{array}{ccc} E_{UAV}^{\pi^{*}}\left(u\right) & = & ∥\xi_{0}-\xi_{5}∥+∥\xi_{5}-\xi_{9}∥+∥\xi_{9}-\xi_{3}∥+∥\xi_{3}-\xi_{10}∥+∥\xi_{10}-\xi_{1}∥+∥\xi_{1}-\xi_{8}∥\\ + & ∥\xi_{8}-\xi_{6}∥+∥\xi_{6}-\xi_{7}∥+∥\xi_{7}-\xi_{0}∥\\ = & \left(\sqrt{8}+\sqrt{10}+\sqrt{10}+\sqrt{125}+\sqrt{2}+\sqrt{5}+\sqrt{5}+\sqrt{34}+\sqrt{5}\right)\times C_{UAV}\\ \approx & 34.29\times C_{UAV} \end{array}$$

The UAV with $B=30\times C_{UAV}$ desists from visiting CH robot 2, CH robot 3, CH robot 4, and CH robot 9 which results in $10+20+\left(13+10\right)+10=63\times C_{CH}$ total energy consumption of CH robots. Thus, the UAV consumes

(34) $$\begin{array}{c} E_{UAV}^{\pi^{*}}\left(u\right)=∥\xi_{0}-\xi_{5}∥+∥\xi_{5}-\xi_{10}∥+∥\xi_{10}-\xi_{1}∥+∥\xi_{1}-\xi_{8}∥+∥\xi_{8}-\xi_{6}∥+∥\xi_{6}-\xi_{7}∥+∥\xi_{7}-\xi_{0}∥\\ =\left(\sqrt{8}+\sqrt{145}+\sqrt{2}+\sqrt{5}+\sqrt{5}+\sqrt{34}+\sqrt{5}\right)\times C_{UAV}\approx 28.83\times C_{UAV} \end{array}$$

The UAV with $B=25\times C_{UAV}$ desists from visiting CH robot 2, CH robot 3, CH robot 4, CH robot 5, and CH robot 9 which results in $\left(10+8\right)+\left(20+8\right)+\left(13+10+8\right)+8+\left(10+8\right)=103\times C_{CH}$ total energy consumption of CH robots. Thus, the UAV consumes

(35) $$\begin{array}{ccc} E_{UAV}^{\pi^{*}}\left(u\right) & = & ∥\xi_{0}-\xi_{10}∥+∥\xi_{10}-\xi_{1}∥+∥\xi_{1}-\xi_{8}∥+∥\xi_{8}-\xi_{6}∥+∥\xi_{6}-\xi_{7}∥+∥\xi_{7}-\xi_{0}∥\\ = & \left(\sqrt{85}+\sqrt{2}+\sqrt{5}+\sqrt{5}+\sqrt{34}+\sqrt{5}\right)\times C_{UAV}\approx 23.17\times C_{UAV} \end{array}$$

The UAV with $B=20\times C_{UAV}$ desists from visiting CH robot 1, CH robot 2, CH robot 3, CH robot 4, CH robot 5, CH robot 9, and CH robot 10 which results in $5+\left(10+8\right)+\left(20+8\right)+\left(13+10+8\right)+8+\left(10+8\right)+\left(2+5\right)=115\times C_{CH}$ total energy consumption of CH robots. Thus, the UAV consumes

(36) $$\begin{array}{ccc} E_{UAV}^{\pi^{*}}\left(u\right) & = & ∥\xi_{0}-\xi_{8}∥+∥\xi_{8}-\xi_{6}∥+∥\xi_{6}-\xi_{7}∥+∥\xi_{7}-\xi_{0}∥\\ = & \left(\sqrt{80}+\sqrt{5}+\sqrt{34}+\sqrt{5}\right)\times C_{UAV}\approx 19.25\times C_{UAV} \end{array}$$

The UAV with $B=15\times C_{UAV}$ visits only CH robot 6 which results in $\left(5+5\right)+\left(10+8\right)+\left(20+8\right)+\left(13+10+8\right)+8+5+\left(10+8\right)+5+\left(2+5+5\right)=135\times C_{CH}$. Thus, the UAV consumes

(37) $$\begin{array}{ccc} E_{UAV}^{\pi^{*}}\left(u\right) & = & ∥\xi_{0}-\xi_{6}∥+∥\xi_{6}-\xi_{0}∥\\ = & \left(\sqrt{53}+\sqrt{53}\right)\times C_{UAV}\approx 14.56\times C_{UAV} \end{array}$$

The UAV with $B=10\times C_{UAV}$ visits only CH robot 5 and CH robot 7 which results in $\left(5+5+53\right)+\left(10+8\right)+\left(20+8\right)+\left(13+10+8\right)+53+\left(10+8\right)+\left(5+53\right)+\left(2+5+5+53\right)=334\times C_{CH}$ total energy consumption of CH robots. Thus, the UAV consumes

(38) $$\begin{array}{ccc} E_{UAV}^{\pi^{*}}\left(u\right) & = & ∥\xi_{0}-\xi_{5}∥+∥\xi_{5}-\xi_{7}∥+∥\xi_{7}-\xi_{0}∥\\ = & \left(\sqrt{8}+\sqrt{17}+\sqrt{5}\right)\times C_{UAV}\approx 9.19\times C_{UAV} \end{array}$$

The UAV with $B=5\times C_{UAV}$ visit only CH robot 7 which results in $\left(5+5+53\right)+\left(10+8\right)+\left(20+8\right)+\left(13+10+8\right)+8+53+\left(10+8\right)+\left(5+53\right)+\left(2+5+5+53\right)=342\times C_{CH}$ total energy consumption of CH robots. Even with B=5, optimal strategy performs better than no strategy and the UAV-oriented strategy.

Table 6 summarizes indices of the nonvisited CH robots which the strategies decide to desist from visiting in the configuration of Figure 7. Similarly, Table 7 summarizes total energy consumption of the nonvisited CH robots which the strategies decides to desist from visiting. From these tables, it can be observed how UAV decides to desist from visiting a subset of CH robots depending on its battery capacity in the configuration of Figure 7. Furthermore, the total energy consumption of the nonvisited CH robots depends on the desisting decisions taken by UAV and so the battery capacity of the UAV. Besides these, the total energy consumption of the nonvisited CH robots also depends on the network topology, the locations of all CH robots.

We compare the performances of the UAV-oriented strategy and our two-stage optimal strategy for battery capacities varying from B=5 to B=50 in the configuration in Figure 7. From Figure 8, the following observations can be made. Applying the UAV-oriented strategy results in $491\times C_{CH}$ for battery capacities from B=10 to B=50 and it is not applicable for B=5 which is insufficient battery capacity for the UAV to travel any CH robot and turn back to the FC. This strategy performs worse than no strategy by which the UAV standing on the FC collects data from all CH robots (no strategy results in $461\times C_{CH}<491\times C_{CH}$). On the other hand, the two-stage optimal strategy achieves zero energy consumption of all CH robots for B=45 and B=50. Optimal strategy results in $10\times C_{CH}$ total energy consumption, only 2% of that by UAV-Oriented strategy for B=40. Optimal strategy results in $23\times C_{CH}$, less than 5% of that by UAV-Oriented strategy for B=35. For B=30, optimal strategy results in $63\times C_{CH}$, less than 13% of that by UAV-Oriented strategy. For B=25, B=20 and B=15, optimal strategy results in $103\times C_{CH}$, $115\times C_{CH}$ and $135\times C_{CH}$ total energy consumption, respectively, still less than 28% of that by the UAV-oriented strategy. For B=10, optimal strategy results in $334\times C_{CH}$, nearly 68% of that by the UAV-oriented strategy. Even for B=5, optimal strategy results in $342\times C_{CH}$ total energy consumption, still less than 75% of that by no strategy. Remember that the UAV-oriented strategy is not applicable for B=5.

## 7. Conclusions

In this work, we investigate a data collection problem by a mobile sink, an unmanned aerial vehicle (UAV) with limited battery capacity, in a robot network divided into several robot clusters. In each cluster, a cluster head (CH) robot allocates tasks to the remaining robots in the cluster and collects data from them and then transmit data to the UAV directly or indirectly (by sending its data to another CH robot to forward to the UAV). In this network, UAV visits some of the CH robots or all of them depending on their locations and the battery capacity of the UAV. If the UAV cannot visit all of CH robots due to the limited battery capacity, then the CH robots not visited by the UAV transmits their data to one of the neighbor CH robot. The aim of the UAV is to minimize the total energy consumption of the CH robots by planning a path.

We propose a two-stage solution for this problem. first, we consider the problem as a traveling salesman problem (TSP) by taking unlimited battery capacity for the UAV. In the second stage, we remove some of the CH robots from the path in order to reduce the energy consumption of the UAV upto the battery capacity of the UAV. We handle the problem by using an analytical approach and obtain the optimal strategy for this problem. Our strategy is compared with the approaches in the close literature for varying number of clusters. The numerical results show that our approach performs much better than the approach in the close literature for various number of CH robots and various battery capacities of the UAV. Hence, our strategy minimizes the total energy consumption of the CH robots optimally depending on the locations of the CH robots and the battery capacity of the UAV.

In our work, we consider the amount and accuracy of all data from all CH robots equally; however, the amount and accuracy of data from different CH robots may differ due to many factors such as difference in the performance of robots and sensors. Therefore, in future work, we plan to consider the problem with varying amount of data for different CH robots. In this scenario, the UAV also evaluates the decision efficiencies of the CH robots which allocates tasks to the other robots. The problem can also be considered under the assumption that visiting a certain specific portion of CH robots are mandatory for the UAV. As the intensity of the acquisition signal can be reduced due to forwarding, this will be our consideration of the near future. This will possibly include the relay cost, i.e., the energy consumed by the CH robot to forward the data received by other CH robot/s.

## Figures and Tables

**Table 1:** Symbols and Notation

Places	Explanations
*M*	The number of all CH robots
*S*	The index set of all CH robots
$\xi_{i}$	The position of the CH robot *i*
$\xi_{0}$	The initial position of the UAV
$E_{UAV}\left(i,j\right)$	The energy consumed by the UAV for the path from CH robot *i* to CH robot *j*
$C_{UAV}$	The constant ratio between the energy consumption of the UAV and the distance it travels
$E_{CH}\left(i,j\right)$	The energy consumed by the CH robot *i* to transmit data to CH robot *j*
$C_{CH}$	The ratio between energy consumption of a CH robot and the square of length of the path
	it forwarded its data
$\pi (\xi_{i},\xi_{j})$	The linear path from CH robot *i* to CH robot *j*
*P*	The set of all possible paths between the CH robots
π	The strategy, the set of the linear paths followed by the UAV for the data collection
$E_{UAV}^{\pi }$	Energy consumption of the UAV under strategy π
$u_{i}$	The data forwarding strategy of CH robot *i* not visited by the UAV
*u*	The set of all data forwarding strategies for all CH robots
$E_{i}^{\pi }\left(u_{i}\right)$	Energy consumption of CH robot *i* under strategies π, $u_{i}$
$E_{ACH}^{\pi }\left(u_{i}\right)$	Energy consumption of all CH robots under strategies π, *u*

**Table 2:** The table shows indices of the nonvisited CH robots depending on battery capacity of UAV in Figure 3. “None” implies that UAV visits all CH robots if B=45 or B=50. “×” implies that the UAV-oriented Strategy is infeasible for that battery capacity.

Strategy	B = 5	B = 10	B = 15	B = 20	B = 25	B = 30	B = 35	B = 40	B = 45	B = 50
UAV-Oriented Strategy	×	1, 3–5	1, 3–5	1, 3–5	1, 3–5	1, 3–5	1, 3–5	1, 3–5	1, 3–5	1, 3–5
Optimal Strategy	1–5	1–3,5	1,3,5	2–5	2,3,5	3,5	3	3	None	None

**Table 3:** The table shows total energy consumption of nonvisited CH robots depending on battery capacity of UAV in Figure 4. “×” implies that the UAV-oriented strategy is infeasible for that battery capacity.

Strategy	B = 5	B = 10	B = 15	B = 20	B = 25	B = 30	B = 35	B = 40	B = 45	B = 50
UAV-Oriented Strategy	×	328	328	328	328	328	328	328	328	328
Optimal Strategy	224	198	182	135	109	93	80	80	0	0

**Table 4:** The table shows indices of the nonvisited CH robots depending on battery capacity of UAV in Figure 5. “None” implies that the UAV visits all CH robots if B=35,40,45,50. “×” implies that the UAV-oriented strategy is infeasible for that battery capacity.

Strategy	B = 5	B = 10	B = 15	B = 20	B = 25	B = 30	B = 35	B = 40	B = 45	B = 50
UAV-Oriented Strategy	×	1, 2, 4–7	1, 2, 4–7	1–5, 7	1–5, 7	1–5, 7	1–5, 7	1–5, 7	1–5, 7	1–5, 7
Optimal Strategy	1–7	1, 2, 4–6	1, 2, 4–6	1, 2, 4, 5	2, 5	5	None	None	None	None

**Table 5:** The table shows total energy consumption of nonvisited CH robots depending on battery capacity of UAV in Figure 6. “×” implies that the UAV-oriented strategy is infeasible for that battery capacity.

Strategy	B = 5	B = 10	B = 15	B = 20	B = 25	B = 30	B = 35	B = 40	B = 45	B = 50
UAV-Oriented Strategy	×	213	213	142	142	142	142	142	142	142
Optimal Strategy	234	173	173	74	51	34	0	0	0	0

**Table 6:** The table shows indices of the nonvisited CH robots depending on battery capacity of UAV in Figure 7. “None” implies that the UAV visits all CH robots if B=45 or B=50. Strategy, UAV-oriented Strategy and optimal strategy are denoted by S, U and O, respectively, in this table.

S	B = 5	B = 10	B = 15	B = 20	B = 25	B = 30	B = 35	B = 40	B = 45	B = 50
U	1–6, 8–10	1–6, 8–10	1–6, 8–10	1–6, 8–10	1–6, 8–10	1–6, 8–10	1–6, 8–10	1–6, 8–10	1–6, 8–10	1–6, 8–10
O	1–6, 8–10	1–4, 6, 8–10	1-5,7-10	1-5,9,10	2-5,9	2-4,9	2,4	2	None	None

**Table 7:** The table shows total energy consumption of nonvisited CH robots depending on battery capacity of UAV in Figure 8.

Strategy	B = 5	B = 10	B = 15	B = 20	B = 25	B = 30	B = 35	B = 40	B = 45	B = 50
UAV-Oriented Strategy	491	491	491	491	491	491	491	491	491	491
Optimal Strategy	342	334	135	105	103	63	23	10	0	0

**Figure A1:**
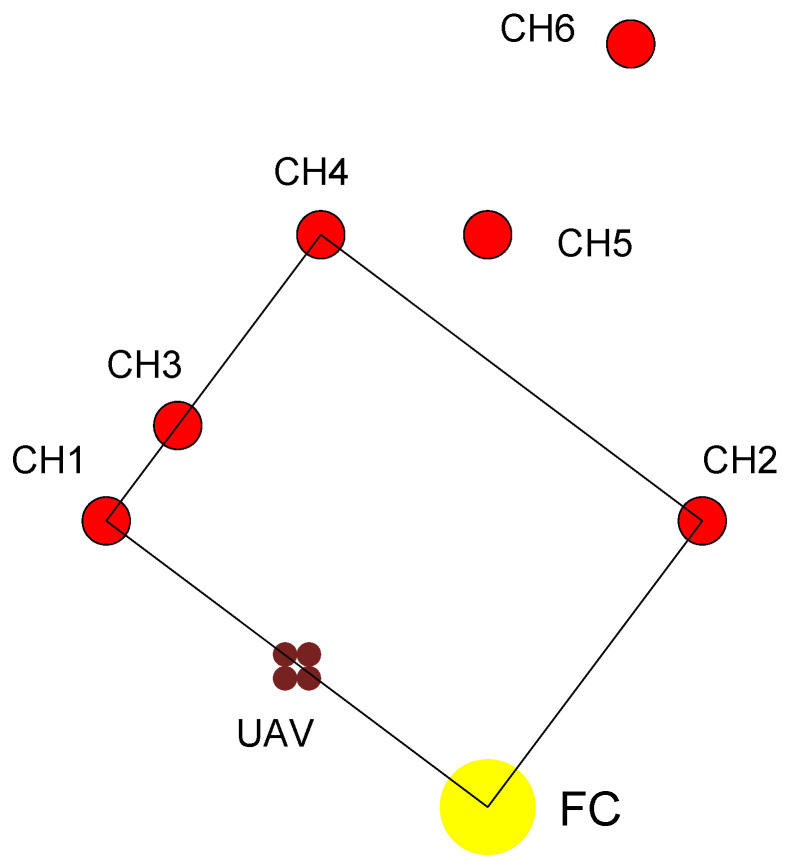
The whole multi-robot system consists of a fusion center (FC) where the UAV starts its route and 6 cluster of robots around FC. The red circles show the locations of the cluster head (CH) robots in their robot cluster. *The UAV uses the strategy $\pi_{1}$ for the data collection*. With respect to this initial position of the UAV at $\xi_{0}=\left(0,0\right)$, the positions of the CH robots are $\left(\xi_{1},\xi_{2},\xi_{3},\xi_{4},\xi_{5},\xi_{6}\right)=\left((-16,12),(9,16),(-13,16),(-7,24),(0,24),(6,32)\right)$ units.

**Figure A2:**
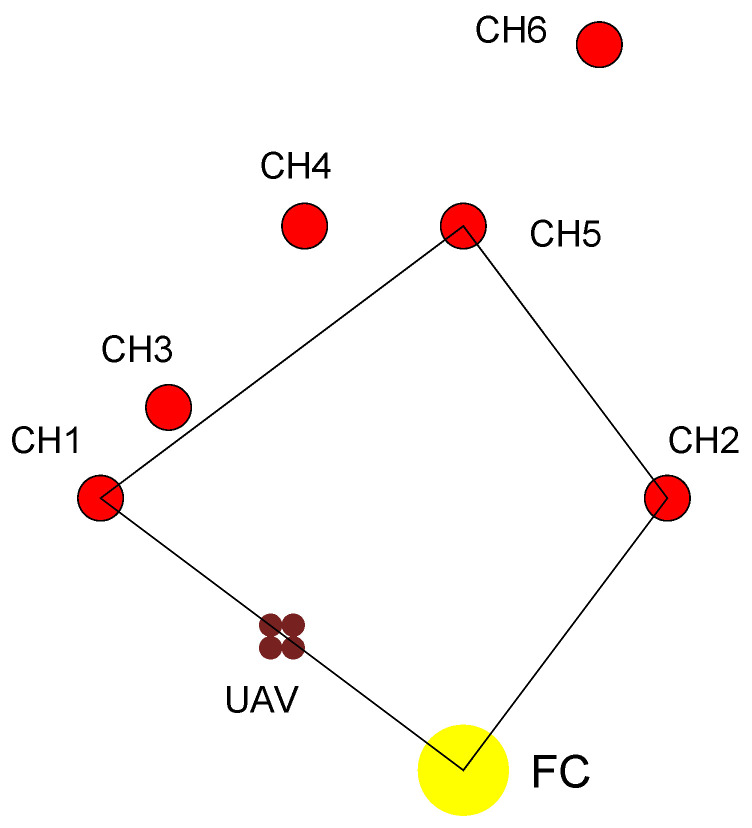
The whole multi-robot system consists of a fusion center (FC) where the UAV starts its route and 6 cluster of robots around FC. The red circles show the locations of the cluster head (CH) robots in their robot cluster. *The UAV uses the strategy $\pi_{2}$ for the data collection*. With respect to this initial position of the UAV at $\xi_{0}=\left(0,0\right)$, the positions of the Ch robots are $\left(\xi_{1},\xi_{2},\xi_{3},\xi_{4},\xi_{5},\xi_{6}\right)=\left((-16,12),(9,16),(-13,16),(-7,24),(0,24),(6,32)\right)$ units.

**Figure A3:**
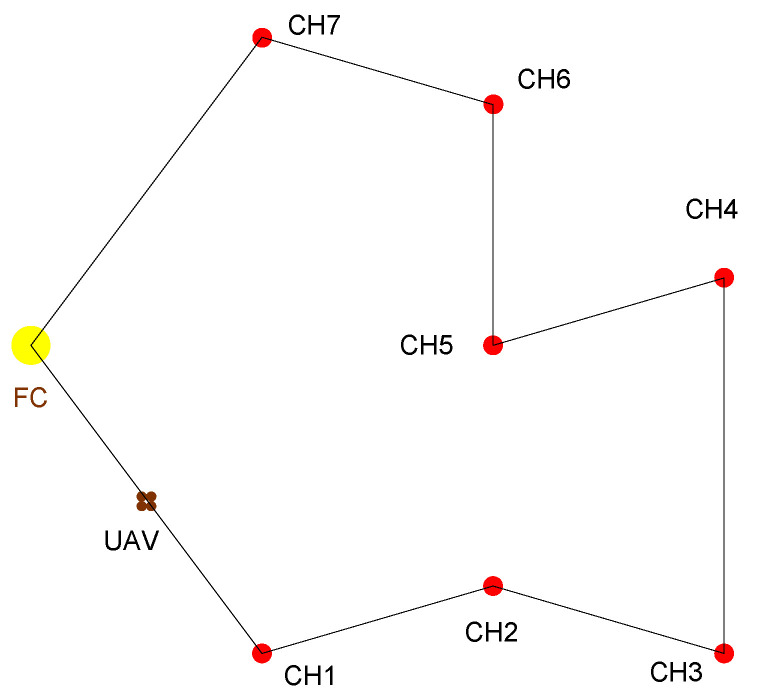
The whole multi-robot system consists of a fusion center (FC) where the UAV starts its route and 7 cluster head (CH) robots around FC. The red circles show the locations of the cluster head (CH) robots in their robot cluster. *The UAV uses an optimal strategy such that it visits CH 1, CH 2, CH 3, CH 4, CH 5, CH 6, and CH 7 in order (The path has a length of 244 m).* With respect to the initial position at $\xi_{0}=\left(0,0\right)$, the positions of the CH robots are $\left(\xi_{1},\xi_{2},\xi_{3},\xi_{4},\xi_{5},\xi_{6},\xi_{7}\right)=\left((24,-32),(48,-25),(72,-32),(72,7),(48,0),(48,25),(24,32)\right)m$.

**Figure A4:**
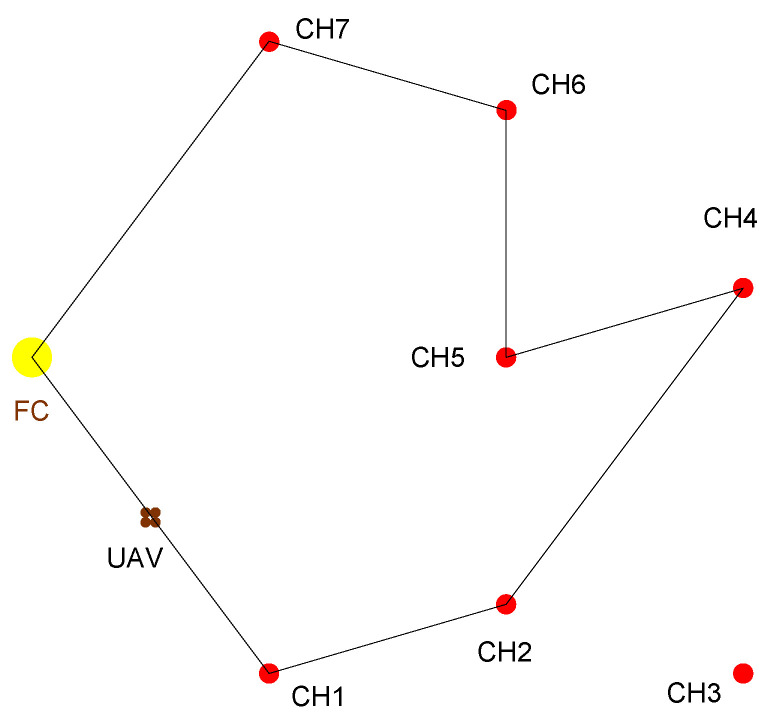
The whole multi-robot system consists of a fusion center (FC) where the UAV starts its route and 7 cluster head (CH) robots around FC. The red circles show the locations of the cluster head (CH) robots in their robot cluster. *The UAV uses an optimal strategy such that it visits CH 1, CH 2, CH 4, CH 5, CH 6, and CH 7 in order (The path has a length of 220 m).* With respect to the initial position at $\xi_{0}=\left(0,0\right)$, the positions of the CH robots are $\left(\xi_{1},\xi_{2},\xi_{3},\xi_{4},\xi_{5},\xi_{6},\xi_{7}\right)=\left((24,-32),(48,-25),(72,-32),(72,7),(48,0),(48,25),(24,32)\right)m$.

**Figure A5:**
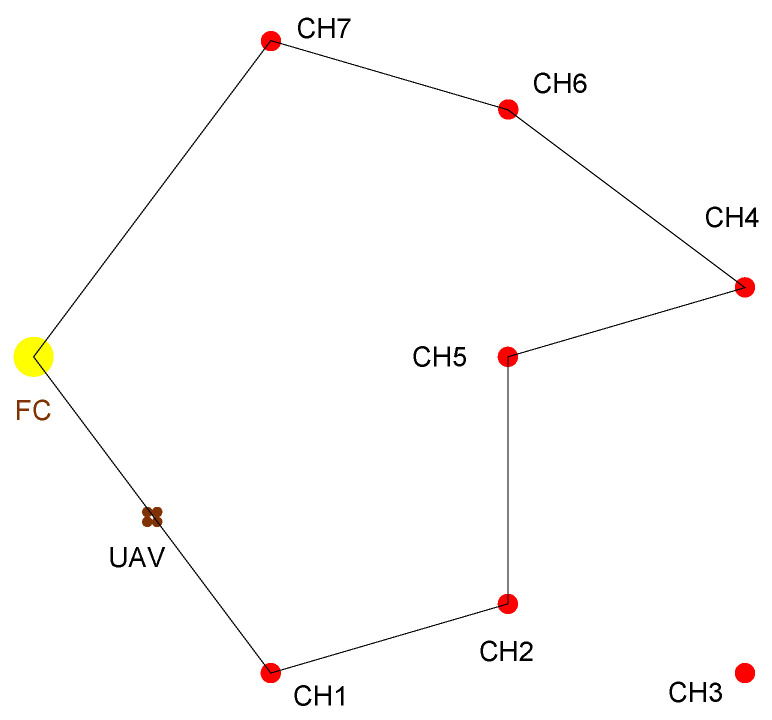
The whole multi-robot system consists of a fusion center (FC) where the UAV starts its route and 7 cluster head (CH) robots around FC. The red circles show the locations of the cluster head (CH) robots in their robot cluster. *The UAV uses an optimal strategy such that it visits CH 1, CH 2, CH 5, CH 4, CH 6 and CH 7 in order (The path has a length of 210 m).* With respect to the initial position at $\xi_{0}=\left(0,0\right)$, the positions of the CH robots are $\left(\xi_{1},\xi_{2},\xi_{3},\xi_{4},\xi_{5},\xi_{6},\xi_{7}\right)=\left((24,-32),(48,-25),(72,-32),(72,7),(48,0),(48,25),(24,32)\right)m$.

## Appendix A. Proof of Proposition 1

This proposition will be proved by a counter example system in Figure A1 and Figure A2.

In this example, we take the initial position of the UAV as $\xi_{0}=\left(0,0\right)$. With respect to this initial position, the positions of the CH robots are $\left(\xi_{1},\xi_{2},\xi_{3},\xi_{4},\xi_{5},\xi_{6}\right)=\left((-16,12),(9,16),(-13,16),(-7,24),(0,24),(6,32)\right)$.

With respect to this positions, we obtain the distance between the CH robots. $∥\xi_{1}-\xi_{3}∥=5$, $∥\xi_{3}-\xi_{4}∥=10$, $∥\xi_{4}-\xi_{5}∥=7$, $∥\xi_{5}-\xi_{2}∥=15$, $∥\xi_{6}-\xi_{5}∥=10$, $∥\xi_{1}-\xi_{5}∥=20$, $∥\xi_{0}-\xi_{1}∥=20$, $∥\xi_{0}-\xi_{2}∥=15$ where $\xi_{0}$ is the initial point of route (trajectory) of UAV.

Assume that B=70 and let’s investigate the cost of the following two strategies, $\pi_{1}$ as shown in Figure A1 and $\pi_{2}$ as shown in Figure A2.

(A1) $$\begin{array}{ccc} \pi_{1} & = & \left\{p\left(\xi_{0},\xi_{1}\right),p\left(\xi_{1},\xi_{3}\right),p\left(\xi_{3},\xi_{4}\right),p\left(\xi_{4},\xi_{2}\right),p\left(\xi_{2},\xi_{0}\right)\right\} \end{array}$$

(A2) $$\begin{array}{ccc} \pi_{2} & = & \left\{p\left(\xi_{0},\xi_{1}\right),p\left(\xi_{1},\xi_{5}\right),p\left(\xi_{5},\xi_{2}\right),p\left(\xi_{2},\xi_{0}\right)\right\} \end{array}$$

The UAV consumes the following energies under these two strategies.

(A3) $$\begin{array}{ccc} E_{UAV}^{\pi_{1}} & = & ∥\xi_{0}-\xi_{1}∥+∥\xi_{1}-\xi_{3}∥+∥\xi_{3}-\xi_{4}∥+∥\xi_{4}-\xi_{2}∥+∥\xi_{2}-\xi_{0}∥ \end{array}$$

(A4) $$\begin{array}{ccc} E_{UAV}^{\pi_{2}} & = & ∥\xi_{0}-\xi_{1}∥+∥\xi_{1}-\xi_{5}∥+∥\xi_{5}-\xi_{2}∥+∥\xi_{2}-\xi_{0}∥ \end{array}$$

From Equations (A3) and (A4), $E_{UAV}^{\pi_{1}}=E_{UAV}^{\pi_{2}}=70$ which equals to the battery capacity of the UAV, *B*. In this case, $\pi_{1}=\pi_{TSP}^{max}$ because it visits maximum number of CH robots, 4.

Let’s compare the cost of these two strategies $\pi_{1}$ and $\pi_{2}$ for Problem 1.

The total energy consumption of the CH robots under the strategy $\pi_{1}$

(A5) $$\begin{array}{ccc} E_{ACH}^{\pi_{1}} & = & \sum_{i=1}^{6}\left[\gamma_{i}^{\pi_{1}}\left(1-\sum_{j=1}^{6}I_{\left\{p\left(\xi_{i},\xi_{j}\right)\in \pi^{1}\right\}}\right)\right], \end{array}$$

(A6) $$\begin{array}{cc} = & \gamma_{5}^{\pi_{1}}+\gamma_{6}^{\pi_{1}}, \end{array}$$

(A7) $$\begin{array}{cc} = & ∥\xi_{5}-\xi_{4}{∥}^{2}+\left[∥\xi_{5}-\xi_{4}{∥}^{2}+{∥\xi_{6}-\xi_{5}∥}^{2}\right] \end{array}$$

The total energy consumption of the CH robots under the strategy $\pi_{2}$

(A8) $$\begin{array}{ccc} E_{ACH}^{\pi_{2}} & = & \sum_{i=1}^{6}\left[\gamma_{i}^{\pi_{2}}\left(1-\sum_{j=1}^{6}I_{\left\{p\left(\xi_{i},\xi_{j}\right)\in \pi_{2}\right\}}\right)\right], \end{array}$$

(A9) $$\begin{array}{cc} = & \gamma_{3}^{\pi_{1}}+\gamma_{4}^{\pi_{1}}+\gamma_{6}^{\pi_{1}}, \end{array}$$

(A10) $$\begin{array}{cc} = & ∥\xi_{3}-\xi_{1}{∥}^{2}+∥\xi_{5}-\xi_{4}{∥}^{2}+{∥\xi_{6}-\xi_{5}∥}^{2} \end{array}$$

From Equations (A7) and (A10),

(A11) $$\begin{array}{ccc} E_{ACH}^{\pi_{1}}-E_{ACH}^{\pi_{2}} & = & ∥\xi_{5}-\xi_{4}{∥}^{2}-{∥\xi_{3}-\xi_{1}∥}^{2}\\ E_{ACH}^{\pi_{1}} & > & E_{ACH}^{\pi_{2}} \end{array}$$

As it can be seen from Figure A1, $\pi_{2}=\pi^{*}$ for Problem 1. Remind that $\pi_{1}=\pi_{TSP}^{max}$ is the optimal strategy for the TSP which aims to visit the maximum number of CH robots, 4 in this example. From Equation (A11),

(A12) $$E_{ACH}^{\pi_{TSP}^{max}}>E_{ACH}^{*}$$

which yields $\pi_{TSP}^{max}$ cannot achieve optimality in this example. Hence, it is proved.

## Appendix B. Proof of Lemma 1

The proof will be done by contradiction. Consider a multi-robot system in Figure A3 which consists of a fusion center (FC) where the UAV starts its route and 7 cluster head (CH) robots around FC. The red circles show the locations of the cluster head (CH) robots in their robot cluster (to focus on the CH robots, the other robots are not shown in Figure A3, Figure A4 and Figure A5). It is assumed that the initial position of the UAV, the position of the fusion center is $\xi_{0}=\left(0,0\right)$. With respect to this initial position, the positions of the CH robots are $\left(\xi_{1},\xi_{2},\xi_{3},\xi_{4},\xi_{5},\xi_{6},\xi_{7}\right)=\left((24,-32),(48,-25),(72,-32),(72,7),(48,0),(48,25),(24,32)\right)m$. The UAV uses an optimal strategy such that it visits CH 1, CH 2, CH 3, CH 4, CH 5, CH 6 and CH 7 in order. To follow that route, the UAV needs exactly the following amount of energy

(A13) $$\begin{array}{ccc} E_{UAV}^{\pi } & = & E_{UAV}\left(0,1\right)+E_{UAV}\left(1,2\right)+E_{UAV}\left(2,3\right)+E_{UAV}\left(3,4\right)+E_{UAV}\left(4,5\right)+E_{UAV}\left(5,6\right)\\ + & E_{UAV}\left(6,7\right)+E_{UAV}\left(7,0\right)\\ = & C_{UAV}\times [∥\xi_{0}-\xi_{1}∥+∥\xi_{1}-\xi_{2}∥+∥\xi_{2}-\xi_{3}∥+∥\xi_{3}-\xi_{4}∥+∥\xi_{4}-\xi_{5}∥+∥\xi_{5}-\xi_{6}∥\\ + & ∥\xi_{6}-\xi_{7}∥+∥\xi_{7}-\xi_{0}∥]\\ = & C_{UAV}\times \left[40+25+25+39+25+25+25+40\right]=244\times C_{UAV} \end{array}$$

From Equation (A13), if $B<244\times C_{UAV}$, then the UAV need to desist from visiting at least one CH robot. Assume that the UAV decided to desist from visiting CH 3 and then search for an optimal route to visit all CH robots except CH 3.

Consider a multi-robot system in Figure A4 which is the same system in Figure A3.

The UAV uses a strategy such that it visits CH 1, CH 2, CH 4, CH 5, CH 6, and CH 7 in order. In this strategy, instead of visiting CH 3, the UAV visits CH 4 just after visiting CH 2 (reconstructing the route with a direct line from CH 2 to CH 4) To follow that route, the UAV needs exactly the following amount of energy

(A14) $$\begin{array}{cc} lE_{UAV}^{\pi } & =E_{UAV}\left(0,1\right)+E_{UAV}\left(1,2\right)+E_{UAV}\left(2,4\right)+E_{UAV}\left(4,5\right)+E_{UAV}\left(5,6\right)+E_{UAV}\left(6,7\right)+E_{UAV}\left(7,0\right)\\ =C_{UAV}\times [∥\xi_{0}-\xi_{1}∥+∥\xi_{1}-\xi_{2}∥+∥\xi_{2}-\xi_{4}∥+∥\xi_{4}-\xi_{5}∥+∥\xi_{5}-\xi_{6}∥+∥\xi_{6}-\xi_{7}∥+∥\xi_{7}-\xi_{0}∥]\\ =C_{UAV}\times \left[40+25+40+25+25+25+40\right]=220\times C_{UAV} \end{array}$$

Consider a multi-robot system in Figure A5 which is the same system in Figure A3.

The UAV uses a strategy such that it visits CH 1, CH 2, CH 5, CH 4, CH 6, and CH 7 in order. To follow that route, the UAV needs exactly the following amount of energy

(A15) $$\begin{array}{cc} lE_{UAV}^{\pi } & =E_{UAV}\left(0,1\right)+E_{UAV}\left(1,2\right)+E_{UAV}\left(2,5\right)+E_{UAV}\left(5,4\right)+E_{UAV}\left(4,6\right)+E_{UAV}\left(6,7\right)+E_{UAV}\left(7,0\right)\\ =C_{UAV}\times [∥\xi_{0}-\xi_{1}∥+∥\xi_{1}-\xi_{2}∥+∥\xi_{2}-\xi_{5}∥+∥\xi_{5}-\xi_{4}∥+∥\xi_{4}-\xi_{6}∥+∥\xi_{6}-\xi_{7}∥+∥\xi_{7}-\xi_{0}∥]\\ =C_{UAV}\times \left[40+25+25+25+30+25+40\right]=210\times C_{UAV} \end{array}$$

From Equations (A14) and (A15), using a direct line from CH 2 to CH 4 does not provide the optimal route for visiting all CH robots except CH 3. By this contradiction, the lemma is proved.
